# Caspase-3 regulates RNA splicing and mitochondrial dynamics in microglia during Parkinson’s disease

**DOI:** 10.1038/s41419-026-09141-x

**Published:** 2026-08-04

**Authors:** Rafael Medina-Guzmán, Bazhena Bahatyrevich-Kharitonik, Marie-Kim St-Pierre, Sara Márquez-Pérez, Elena Gavilán, Cristóbal Coronel-Guisado, Alicia Flores-Cortes, Rodrigo García-Valiente, Sandro Argüelles, Edel Kavanagh, Yiyi Yang, Tomas Deierborg, José Luis Venero, Cristina González-Aguilera, Bertrand Joseph, Miguel Ángel Burguillos

**Affiliations:** 1 Instituto de Biomedicina de Sevilla (IBiS, Hospital Universitario Virgen del Rocío/CSIC/Universidad de Sevilla), Seville, Spain; 2 Toxicology Unit, Institute of Environmental Medicine, Karolinska Institutet, Stockholm, Sweden; 3 Centro Andaluz de Biología Molecular y Medicina Regenerativa (CABIMER) Consejo Superior de investigaciones científicas, Universidad de Sevilla, Universidad Pablo de Olavide, Seville, Spain; 4 Association of Spanish Scientists in the Netherlands (CENL-SWNL), Amsterdam, the Netherlands; 5 Department of Physiology - Faculty of Biology, University of Seville, Seville, Spain; 6 Experimental Neuroinflammation Laboratory, Department of Experimental Medical Science, Lund University, Lund, Sweden; 7 Department of Biochemistry and Molecular Biology, Faculty of Pharmacy, University of Seville, Seville, Spain

**Keywords:** Microglia, RNA metabolism

## Abstract

Caspase-3, a cysteine-aspartic protease canonically known for its role in apoptotic cell death, has been implicated in several non-apoptotic functions, particularly in microglia, the brain-resident macrophages. These novel functions appear to depend on distinct levels of caspase-3 activation; however, the role of basal caspase-3 activity remains unclear. Here, we show that basal caspase-3 regulates RNA splicing in microglia, and its deficiency in a Parkinson´s disease (PD) model leads to splicing dysregulation. Loss of basal caspase-3 activity also increases double-stranded RNA (dsRNA) accumulation, inducing a type I interferon response. Additionally, caspase-3 deficiency impairs mitochondrial respiration, reduces ATP production, and causes sex-specific mitochondrial abnormalities, predominantly in female microglia. Together, our findings uncover a non-apoptotic, homeostatic role for basal caspase-3 activity in microglia. Its disruption, such as in PD, may drive key molecular features of neuroinflammation and neurodegeneration, positioning caspase-3 as a critical regulator of microglial function in health and disease.

## Introduction

Caspases are a family of endoproteases with high specificity for certain tetrapeptide motifs [1, 2]. While traditionally associated with apoptosis, caspases also play different roles during developmental and post-developmental stages [3, 4]. Nuclear caspase-3 has been implicated in the pathogenesis of Parkinson’s disease (PD), primarily through its role in dopaminergic neuronal death [5, 6]. However, a cytosolic location of caspase-3 in various cell types under ischemic conditions has suggested the possibility of other non-apoptotic functions [7, 8]. Our work and that of others have shown that caspase-3 activity can be triggered by inflammatory stimuli, contributing to microglial activation in different diseases [9–15], as well as during physiological conditions affecting microglial phagocytic capacity [16, 17] without causing apoptosis. In PD, we observed that inhibition of caspase-8 (an initiator caspase that may process and activate caspase-3), decreased microglial inflammatory response and induced neuroprotection in a murine model of PD induced by intraperitoneal injection with 1-Methyl-4-phenyl-1,2,3,6-tetrahydropyridine (MPTP) [9, 18].

PD is characterized by several pathological hallmarks, including type I interferon (IFN) signaling [19, 20], dysregulation of RNA splicing [21] and mitochondrial dysfunction [22–24]. Type I interferon (IFN) responses are increasingly recognized as key drivers of neurodegeneration, particularly in Parkinson’s disease (PD) [19, 20, 25–31], where inhibition of TANK-binding kinase 1 (TBK1) confers neuroprotection in PD models [25, 32, 33].

Spliceosome activity dysregulation is an emerging area within PD [34–37]. Early reports in PD patients show how RNA splicing factors, such as SRRM2, are consistently dysregulated not only in brain tissue but also in blood samples, suggesting systemic splicing alterations [38]. These abnormal splicing events have been reported to affect genes directly implicated in PD pathology (for instance DJ-1) in PD patients [39]. A recent meta-analysis of transcriptomics and proteomics studies reveals widespread alternative splicing dysregulation in Parkinson’s disease, including differential isoform usage, extended UTRs, and non-coding isoform upregulation, linked to ER stress signaling via IRE1–XBP1 and RIDD pathways, suggesting a critical role for post-transcriptional regulation in PD pathogenesis [36].

Dysregulated RNA splicing can lead to the formation and accumulation of aberrant double-stranded RNA (dsRNA) intermediates [40, 41], which are recognized as contributors to neuroinflammation and neurodegeneration in PD [42]. Conversely, mitochondrial impairment, particularly decreased ATP production, can disrupt ATP-dependent splicing processes, further exacerbating splicing defects [43, 44].

Despite the well-established role of caspase-3 in apoptosis, its basal, non-apoptotic activity in microglia remains poorly understood, particularly with regard to sex-specific effects that have been shown under cell death conditions (for instance, after ischemic insult) [45–48]. Similarly, there is also a clear sex-dependent bias in PD, where males are more affected than females at a ratio of 2:1. Furthermore, in PD, males presented increased gene expression in mechanisms related to metal homeostasis, lipid metabolism, and immunity, while females exhibited mitochondrial and lysosomal dysfunction and alterations to cytoskeletal proteins and glutamic metabolism [49]. Interestingly, in females, though they have higher mitochondrial respiration and ATP production than males [50], lysosomal dysfunction could lead to accumulation of defective mitochondria, further increasing oxidative stress. Here, we uncover a previously unrecognized role for basal caspase-3 activity in maintaining mitochondrial function and RNA splicing homeostasis in microglia. Inhibition of this activity leads to mitochondrial dysfunction, accumulation of double-stranded RNA, and activation of a type I interferon response (molecular signatures observed in PD). These findings identify basal caspase-3 activity as a potential regulator of several key pathways governing microglial biology, and its inhibition can cause an immune response per se.

## Results

### Microglial caspase-3 regulates nigrostriatal pathway integrity in a sex-specific manner in the MPTP mouse model of Parkinson’s disease

**Fig. 1:**
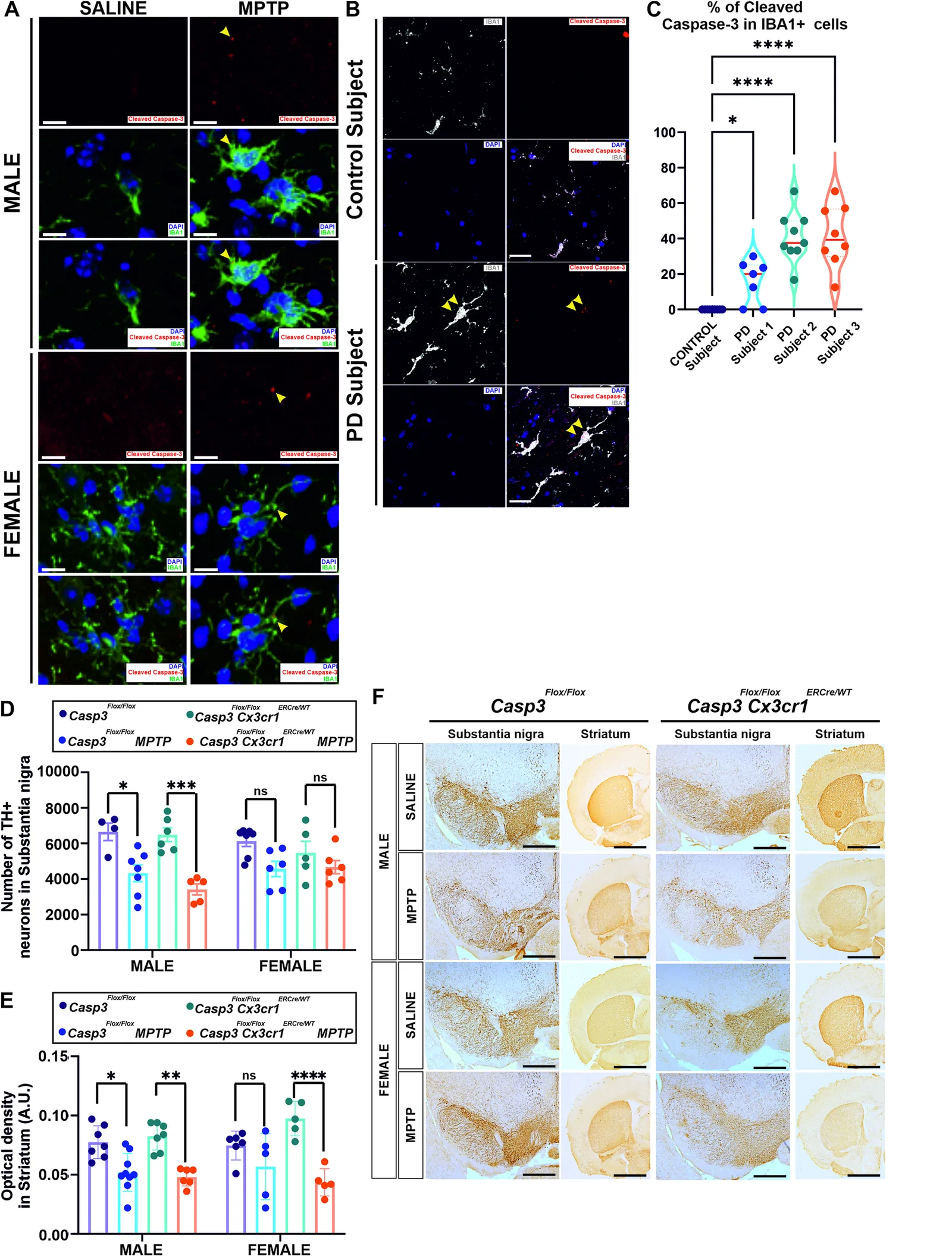
Microglial caspase-3 deficiency augments MPTP-induced neurodegeneration in female mice. **A** Representative confocal images showing cytosolic cleaved caspase-3 (red) within IBA1⁺ cells (green) in the SN of male and female mice. Nuclei counterstained with DAPI (blue). Yellow arrowheads indicate co-localization of cleaved caspase-3 within IBA1⁺ cells (scale bar, 25 µm). **B** Representative confocal images showing cytosolic cleaved caspase-3 (red) within IBA1⁺ cells (green) in the SN of Control and PD subjects. Nuclei counterstained with DAPI (blue). Yellow arrowheads indicate co-localization of cleaved caspase-3 within IBA1⁺ cells (scale bar, 100 µm). **C** Analysis of IBA1+ cells with cleaved caspase-3 in the cytosol in control and PD subjects. Each dot represents independent images taken per subject in the SN (*n* = 7–8). **D** Stereological quantification of TH⁺ neurons in the SN of male and female mice across genotypes and treatments. Each dot represents an individual mouse (*n* = 4–7). **E** Optical density analysis of TH⁺ striatal terminals; each dot represents an individual mouse (*n* = 4–9). **F** Representative TH immunostaining in the SN (**D**) and striatum (**E**) from male and female *Casp3*^*flox/flox*^ and *Casp3*^*flox/flox*^*Cx3cr1*^*CreERT2/WT*^ mice treated with MPTP or saline (scale bar, 100 µm). Data in (**C**) is mean value. Data in (**D**) and (**E**) are mean ± SD. Statistics: one-way ANOVA with Tukey’s *post hoc* test (**C**); three-way ANOVA with Tukey’s *post hoc* test (**D**, **E**). *p < 0.05, **p < 0.01, ***p < 0.001, ****p < 0.001*, ns* statistically non-significant. See also Supplementary Fig. S1.

To investigate the role of caspase-3 in modulating microglial neuroinflammatory responses during Parkinson’s disease (PD), we employed the well-established in vivo MPTP model. Building upon our previous work where caspase-8 inhibition confers neuroprotection in the MPTP model [9, 18], we sought to determine whether microglial caspase-3 deficiency similarly modulates neurodegeneration. First, we assessed the expression of cleaved caspase-3, the active form of the enzyme, in IBA1+ microglia under both saline and MPTP-treated conditions. Immunohistochemical analysis revealed that only a very minor subset of microglia had low expression levels of cleaved caspase-3 following MPTP exposure (Fig. 1A, Fig. S1A), while no activation was detectable under saline conditions (data not shown). Similarly, we analyzed the activation of microglial caspase-3 in a small cohort of PD subjects and a control subject, and observed that between 20 and 40% of microglia expressed cleaved caspase-3 in their cytosol, while we could not observe any expression in an age-matched control (Fig. 1B, C and Fig. S1B).

To explore potential sex-specific roles of microglial caspase-3 in PD-related neurodegeneration, we used transgenic mice harboring an inducible, microglia-specific deletion of caspase-3 (*Casp3*^flox/flox^*Cx3cr1*^CreERT2/WT^). Recombination efficiency was confirmed by EGFP expression following excision of caspase-3 exon 2 (Fig. S1C–E). RT-qPCR analysis of microglia isolated from the substantia nigra (SN) confirmed efficient caspase-3 depletion at the mRNA level in *Casp3*^flox/flox^*Cx3cr1*^CreERT2/WT^ mice compared to *Casp3*^flox/flox^ controls (Fig. S1F).

Stereological quantification of tyrosine hydroxylase (TH)-positive neurons in the SN revealed a sex-dependent effect. In female mice, MPTP treatment did not significantly affect TH+ neuron numbers in either control or caspase-3-deficient microglia mice. In contrast, male mice exhibited a significant reduction in TH+ neurons following MPTP treatment, regardless of microglial caspase-3 status (Fig. 1D, F). Similarly, TH optical density analysis in the striatum showed that MPTP treatment led to a significant reduction in TH expression in males, independent of caspase-3 status. Interestingly, in female *Casp3*^flox/flox^ control mice, MPTP treatment did not reduce striatal TH levels. However, in female *Casp3*^flox/flox^*Cx3cr1*^CreERT2/WT^ mice, MPTP exposure resulted in a significant decrease in TH expression (Fig. 1E, F). These data suggest that microglial caspase-3 deficiency sensitizes female mice to MPTP-induced neurotoxicity, whereas in males, caspase-3 status does not modulate susceptibility. As the MPTP model induces retrograde neurodegeneration [51], this helps explain the absence of cell body degeneration in female *Casp3*^flox/flox^*Cx3cr1*^CreERT2/WT^ mice. Therefore, we decided to investigate in more depth the caspase-3-dependent mechanisms related to microglia upon MPTP treatment.

### Loss of microglial caspase-3 alters cell cycle and type I interferon–related gene expression after MPTP treatment

**Fig. 2:**
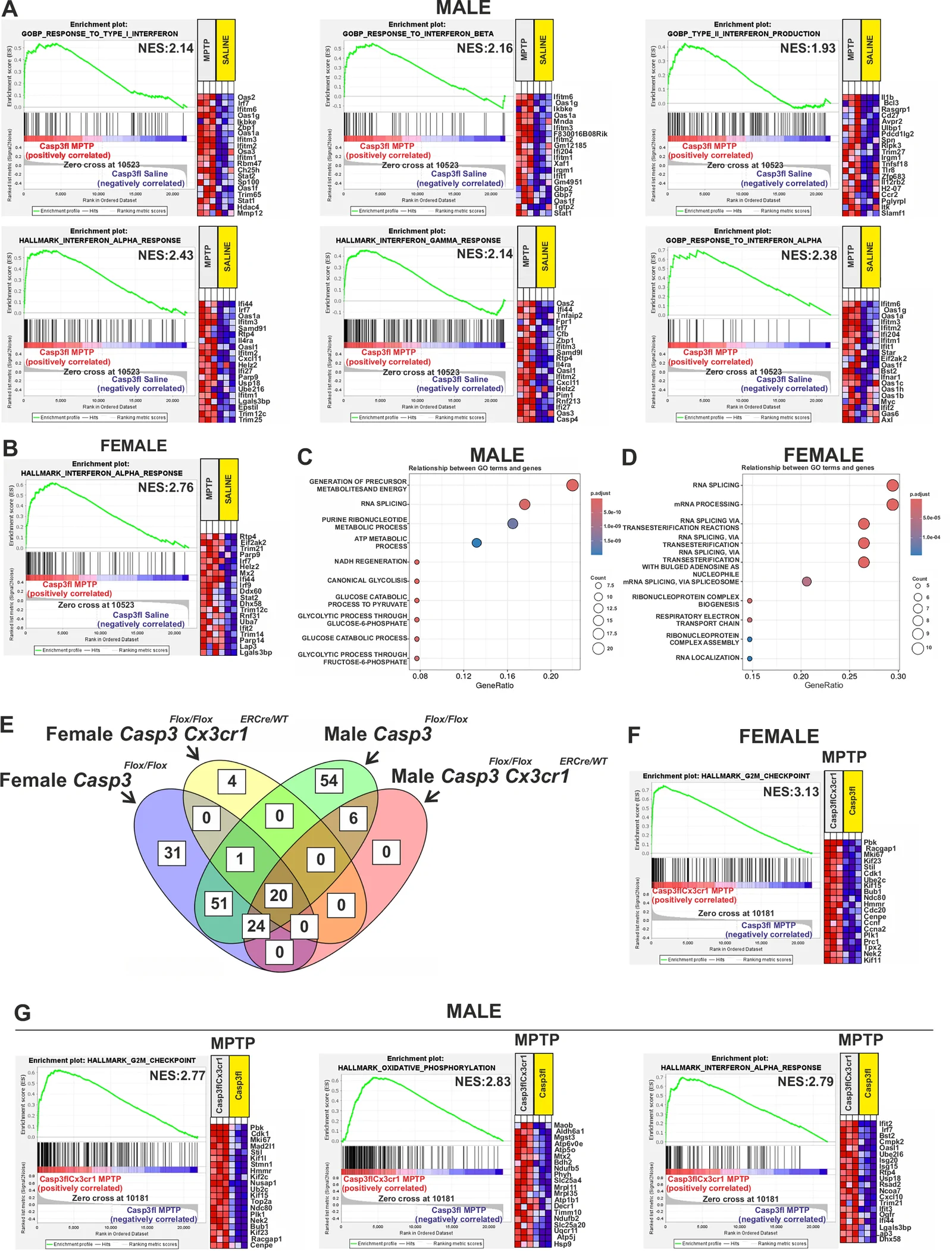
Microglial caspase-3 deficiency alters inflammatory gene expression and protein abundance related to RNA splicing and energy metabolism upon MPTP treatment in a sex-dependent manner. Gene set enrichment analysis showing normalized enrichment scores (NES) and top 20 differentially expressed genes in microglia isolated from the substantia nigra (SN) of male (**A**) and female (**B**) *Casp3*^*flox/flox*^ mice treated with MPTP *versus* saline (*n* = 3 mice per group). Gene Ontology (GO) enrichment dot plots of proteomic data comparing protein abundance in microglia isolated from the SN of male (**C**) and female (**D**) *Casp3*^*flox/flox*^ mice treated with MPTP versus saline. Proteins included were detected in both replicates, annotated with ≥2 unique peptides (*n* = 2 mice per group). **E** Venn diagram illustrating shared and unique proteins identified by proteomic analysis (presence/absence) in microglia from MPTP-treated *Casp3*^*flox/flox*^*Cx3cr1*^*CreERT2/WT*^ and *Casp3*^*flox/flox*^ male and female mice (*n* = 2 mice per group; proteins annotated with ≥2 unique peptides and present in both replicates). Gene set enrichment analysis showing NES values and top 20 differentially expressed genes in microglia from the SN of female (**F**) and male (**G**) MPTP-treated *Casp3*^*flox/flox*^*Cx3cr1*^*CreERT2/WT*^ versus *Casp3*^*flox/flox*^ mice (*n* = 3 mice per group). See also Supplementary Fig. S2.

To characterize microglial responses to MPTP exposure at both transcriptomic and proteomic levels, we isolated microglia from the SN of male and female *Casp3*^flox/flox^ mice treated with either saline or MPTP. Differential gene expression analysis using Transcriptome Analysis Console (TAC) software revealed a small number of significantly altered genes (FDR < 0.05) in MPTP-treated males, including *Cd44*, *Itgal*, *Il1β*, *Oas2* and *Plac8* (Fig. S2A). Interestingly, no differentially expressed genes were detected in females using the same threshold (Fig. S2B). Since MPTP-induced neuroinflammation is likely driven by coordinated changes across entire signaling pathways rather than large shifts in the expression of individual genes, we performed Gene Set Enrichment Analysis (GSEA) on the array data. GSEA revealed that MPTP treatment significantly upregulated pathways related to type I and II IFN responses in male microglia. These included enrichment in gene sets such as GOBP Response to Type I IFN, GOBP Response to IFN-β, GOBP Type II IFN Production, GOBP Response to IFN-α, HALLMARK Interferon α Response, and HALLMARK Interferon γ Response (Fig. 2A). In contrast, female microglia exhibited a more restricted response, with an enrichment only observed for the *HALLMARK Interferon α Response* gene set (Fig. 2B). These findings are consistent with previous studies highlighting the role of type I IFN signaling in the MPTP model of PD [32].

In addition to this, we investigated proteomic changes following MPTP exposure. Microglial protein extracts from the SN were prepared for mass spectrometry, and proteins were identified based on the detection of at least two unique peptides in both biological replicates. Comparative analysis revealed increased abundance of proteins associated with energy metabolism and RNA splicing pathways following MPTP treatment, in males (Fig. 2C) while in females, proteins were predominantly associated with RNA splicing processes (Fig. 2D). In both male and female microglia treated with MPTP, regardless of caspase-3 status, we identified a common cluster of 20 proteins linked to nucleosome assembly (a process essential for DNA packaging, chromatin organization, and gene regulation) and to the generation of precursor metabolites and energy (Fig.2E, Fig. S2C, D). Also, in *Casp3*^*flox/flox*^ mice, 54 proteins were expressed exclusively in males, associated with energy metabolism and splicing, while 31 specific proteins were observed only in *Casp3*^*flox/flox*^ female mice, linked to splicing (Fig. S2D). As a matter of fact, there are no specific proteins identified in male *Casp3*^flox/flox^*Cx3Cr1*^CREERT2/WT^ treated mice and only 4 proteins (APOA1, GAPDHS, H2BC1 and SLC25A31) in female *Casp3*^flox/flox^*Cx3Cr1*^CREERT2/WT^ mice (Fig. S2C).

Afterward, the impact of microglial caspase-3-deficiency response to MPTP treatment was assessed in both male and female mice at gene expression level. The GSEA results showed an increased expression of genes associated with the Hallmark G2/M checkpoint in both genders, and the Hallmarks of Interferon-α response and oxidative phosphorylation in males only (Fig. 2F, G). Interestingly, in *Casp3*^flox/flox^*Cx3Cr1*^CREERT2/WT^ mice, the number of genes affected by MPTP treatment was greater than in *Casp3*^*flox/flox*^ mice (Fig. S2E, F), with many of the genes related to the control of the cell cycle (in both genders) and type I IFN response (only in males) (Fig. S2G, H), in concordance with GSEA results.

### Loss of basal microglial caspase-3 activity promotes sex-dependent changes in cell metabolism and type I interferon response

**Fig. 3:**
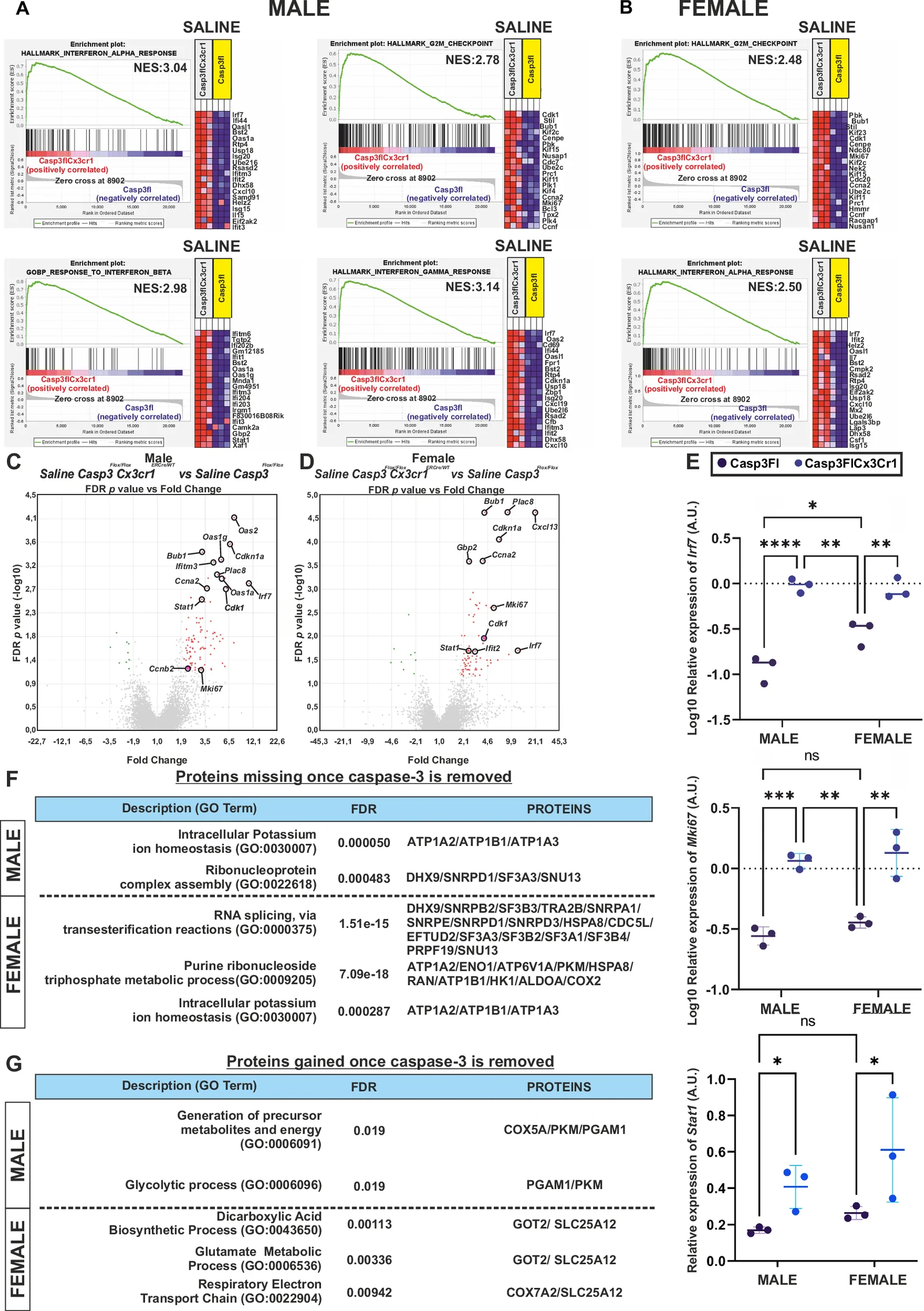
Microglial caspase-3 deficiency alters type I IFN signaling and protein abundance related to RNA splicing and energy metabolism under saline conditions in a sex-dependent manner. Gene set enrichment analysis (GSEA) showing normalized enrichment scores (NES) and the top 20 differentially expressed genes in microglia isolated from the substantia nigra (SN) of male (**A**) and female (**B**) saline-treated *Casp3*^*flox/flox*^*Cx3cr1*^*CreERT2/WT*^ versus *Casp3*^*flox/flox*^ mice (*n* = 3 mice per group). Volcano plots of differentially expressed genes (FDR < 0.05) in microglia from the SN of male (**C**) and female (**D**) *Casp3*^*flox/flox*^*Cx3cr1*^*CreERT2/WT*^ versus *Casp3*^*flox/flox*^ saline-treated mice (*n* = 3 mice per group). **E** RT-qPCR analysis of *Irf7*, *Mki67* and *Stat1* mRNA levels in microglia isolated from the SN of saline-treated *Casp3*^*flox/flox*^ and *Casp3*^*flox/flox*^*Cx3cr1*^*CreERT2/WT*^ mice. Each dot represents one biological replicate (*n* = 3 mice per group). Protein presence/absence tables showing missing (**F**) and gained (**G**) proteins identified by proteomic analysis in microglia from male and female *Casp3*^*flox/flox*^*Cx3cr1*^*CreERT2/WT*^ versus *Casp3*^*flox/flox*^ mice treated with saline. Only proteins detected in both replicates and annotated with ≥2 unique peptides are shown (*n* = 2 mice per group); associated GO terms are included. Data in (**E**) for *Irf7* and *Mki67*is presented as log10-transformed expression values mean ± SD. Data was log-transformed to meet normality assumptions for statistical analysis. Statistics: two-way ANOVA with Tukey’s *post hoc* test (**E**). *p < 0.05, **p < 0.01, ***p < 0.001, ****p < 0.0001. See also Supplementary Fig. S3.

In our transgenic mouse model, tamoxifen was administered daily for 5 consecutive days, followed by over a two-week waiting period before treatment with either saline or MPTP for an additional 96 h (Fig. S1E). During this waiting period, caspase-3 expression is depleted, influencing microglial phenotype. We previously demonstrated that inhibition of basal caspase-3 in a glioblastoma model shifts microglia toward a tumor-supportive phenotype, resulting in increased brain tumor size within the first week after intracerebral injection of glioma cells [52]. For this reason, we examined the transcriptome of microglia obtained from SN belonging to male and female saline-treated *Casp3*^flox/flox^ and *Casp3*^flox/flox^*Cx3Cr1*^CREERT2/WT^ mice to look for any changes in gene expression. Strikingly, Caspase-3 deficiency led to the upregulation of several MPTP-affected pathways. These included the Hallmark IFN-α response (in both males and females), the Hallmark IFN-γ response, and GO Biological Process (GOBP) response to IFN-β (both restricted to males), as well as the Hallmark G2/M checkpoint (observed in both sexes) (Fig. 3A–D; Fig. S3A, B). We validated the increased expression of *Irf7*, *Mki67, and Stat1* in microglia isolated from males and females in *Casp3*^flox/flox^*Cx3Cr1*^CREERT2/WT^ saline-treated mice by RT-qPCR (Fig.3E), those genes serving as representative genes for the type I IFN response and the Hallmark G2/M checkpoint.

At the proteomic level, caspase-3 deficiency resulted in a decreased abundance of several proteins compared to controls (Fig. 3F; Fig. S3C–G). Notably, proteins involved in intracellular potassium ion homeostasis (ATP1A2, ATP1B1, ATP1A3) were reduced in both sexes. In addition, proteins involved in RNA splicing showed sex-specific patterns: in males, reduced expression of proteins associated with ribonucleoprotein complex assembly (DHX9, SNRPD1, SF3A3, SNUL3). In females, splicing machinery components (especially those involved in transesterification) were also reduced (e.g., DHX9, SNRPB2, SF3B3, TRA2B, SNRPA1, SNRPE, SNRPD1, SNU13, and others) (Fig.3F; Fig. S3C and Fig. S3E–G). Also, proteins related to energy metabolism, such as purine ribonucleoside triphosphate metabolic processes (e.g., ATP1A2, ENO1, ATP6V1A, PKM, HSPA8, RAN, ATP1B1, HK1, ALDOA, COX2), were downregulated only in females (Fig. 3F and Fig. S3G). Conversely, only a small number of proteins were found to be upregulated following caspase-3 loss (Fig. 3G; Fig. S3H–J). In males, these included proteins involved in the generation of precursor metabolites and energy (COX5A, PKM, PGAM1) and glycolysis (PGAM1, PKM). In females, the increased proteins were associated with the dicarboxylic acid biosynthetic process and glutamate metabolism (GOT2, SLC25A12), and the respiratory electron transport chain (COX7A2, SLC25A12).

### Short-term inhibition of caspase-3/7 activity disrupts microglial RNA splicing

**Fig. 4:**
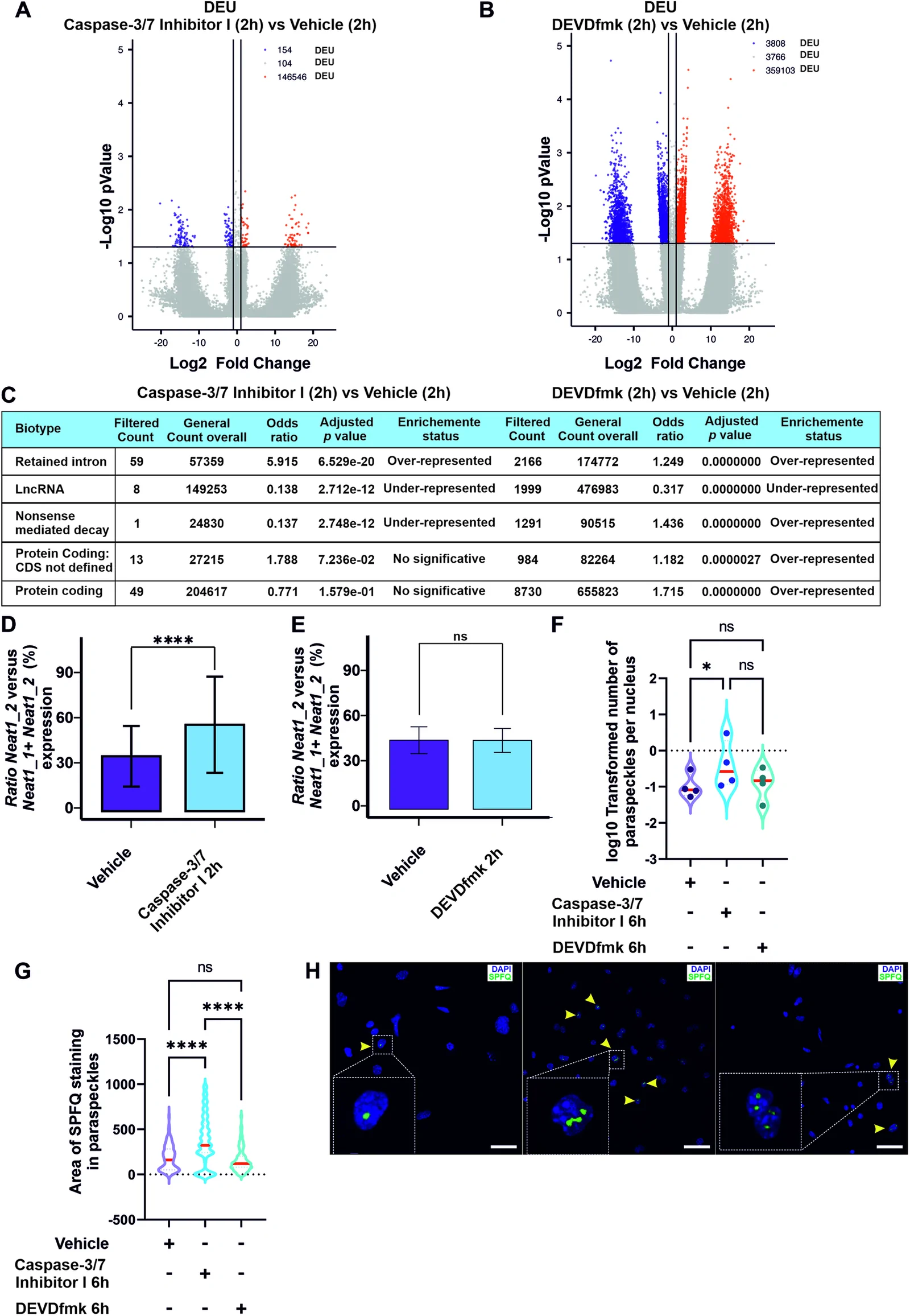
Short-term inhibition of caspase-3/7 alters gene expression and RNA splicing in primary microglia. **A** Volcano plot showing differential exon usage (DEU; *p* < 0.05) from primary microglia treated for 2 h with an allosteric caspase-3/7 inhibitor. **B** Volcano plot showing differential exon usage (DEU; *p* < 0.05) from primary microglia treated for 2 h with an irreversible inhibitor, DEVDfmk. **C** Table summarizing RNA biotypes identified among DEU events shown in (**A** and **B**), including retained introns and other transcript variants. Bar plots showing relative changes in the ratio of NEAT1_2 expression following caspase‑3 inhibition with allosteric (**D**) or DEVDfmk (**E**) based on the data generated from the total RNA-seq in (**A**) and (**B**) (*n* = 4 per group). Violin plots showing quantification of paraspeckle number per nucleus (**F**) and total SFPQ‑positive paraspeckle area per cell (**G**), respectively. **H** Representative confocal images of primary microglia stained for SFPQ (green) and DAPI (blue). Yellow arrowheads indicate SFPQ‑positive nuclear paraspeckles; dashed boxes highlight enlarged nuclei. Scale bars, 100 μm. Data are presented as mean ± SD. Data in (**F**) for the *number of paraspeckles per nucleus is log10-Transformed* is to meet normality assumptions for statistical analysis. Statistics: Fisher´s Test (**D**) and (**E**); Friedman test followed by Dunn’s multiple comparisons test. **F** Mixed‑effects model (REML) with Tukey’s multiple comparisons test (**G**).*p < 0.05, ****p < 0.0001. See also Supplementary Fig. S4.

Our proteomics data in Figs. 2 and 3 suggested that caspase-3 could affect the RNA splicing process, so we next investigated whether caspase-3 could directly regulate it. To this end, primary microglia were treated for 2 h with an allosteric caspase-3/7 inhibitor (Caspase-3/7 Inhibitor I) or with an irreversible caspase-3/7 inhibitor (DEVDfmk), followed by RNA sequencing. To assess the impact on RNA splicing, we performed differential exon usage (DEU) analysis using DEXSeq. Both inhibitors showed enrichment for retained introns, though with different strengths, being more pronounced in the Caspase-3/7 Inhibitor I (allosteric, odds ratio 5.915) than in DEVDfmk (irreversible, odds ratio 1.249) (Fig. 4A–C). We confirm that increased retain intron for the *Ccno* transcript exhibited marked retention of intron 1 upon caspase-3/7 inhibition I (Fig. S4A). The weaker effect of DEVDfmk is likely attributable to the distinct mechanisms of action of the two inhibitors. Under basal conditions, a substantial fraction of caspase‑3 may already be engaged with endogenous substrates, and de novo caspase‑3 activation is expected to be limited. Consequently, competitive active‑site inhibition that occurs with DEVDfmk may have a reduced impact in this context, whereas allosteric inhibition can more effectively modulate caspase‑3 activity. Also, supporting this hypothesis, short-term inhibition with the allosteric inhibitor led to moderate transcriptional changes, with 105 genes significantly upregulated and 29 downregulated (Fig. S4B), while using DEVDfmk did not provoke any statistically different differentially expressed genes (data not shown). One of the genes that was induced with the allosteric inhibitor was the long non‑coding RNA Nuclear Paraspeckle Assembly Transcript 1 (Neat1), known to be a regulator of pre‑mRNA processing [53, 54], as well as an enhancer of IFN‑β production via RIG‑I signaling via paraspeckle synthesis [55]. Curiously, it is the increase in the ratio of the long isoform of Neat1 (Neat1_2, which is 20 kb) vs the short isoform (Neat1_1, which is 3.1 kb; both isoforms share an identical 5′) that promotes the formation of paraspeckles. In our RNA-seq data, we observe an increase in this ratio only in the allosteric inhibitor but not in DEVDfmk (Fig. 4B, C). The increase in total Neat1 expression (Fig. S4C) and the increase in the ratio of the long isoform Neat1_2 (Fig. S4D) were validated by qPCR following the qPCR primer strategy described in Fig. S4E to distinguish between the two isoforms of Neat1. Using the same inhibitors at the same time and dosage in the BV2 microglial cell line to assess the ratio of the long isoform Neat1_2 also showed that the allosteric inhibitor does induce Neat1_2 isoform statistically (Fig. S4H). This increase in the ratio of the long isoform Neat1_2 was also observed when we knocked down caspase-3 expression in BV2 (Fig. S4G), confirming caspase-3′s role in the regulation of Neat1_2 (Fig. S4H).

To determine whether the upregulation of the long NEAT1_2 isoform translated into functional changes, we assessed paraspeckle formation in primary microglia after caspase-3 inhibition. For this reason, primary microglia were treated with both caspase‑3 inhibitors for 6 h, and paraspeckle formation was assessed by immunofluorescence staining of Splicing factor proline‑ and glutamine‑rich (SFPQ), a core structural component of nuclear paraspeckles. (Fig. 4F–H). We confirmed that only the allosteric inhibitor was capable of generating and increasing the number of paraspeckles per cell (Fig. 4F) and the area of such paraspeckles (Fig. 4G).

### Caspase-3 regulates microglial type I IFN response, and interacts with spliceosome and mitochondrial proteins in BV2 cells

**Fig. 5:**
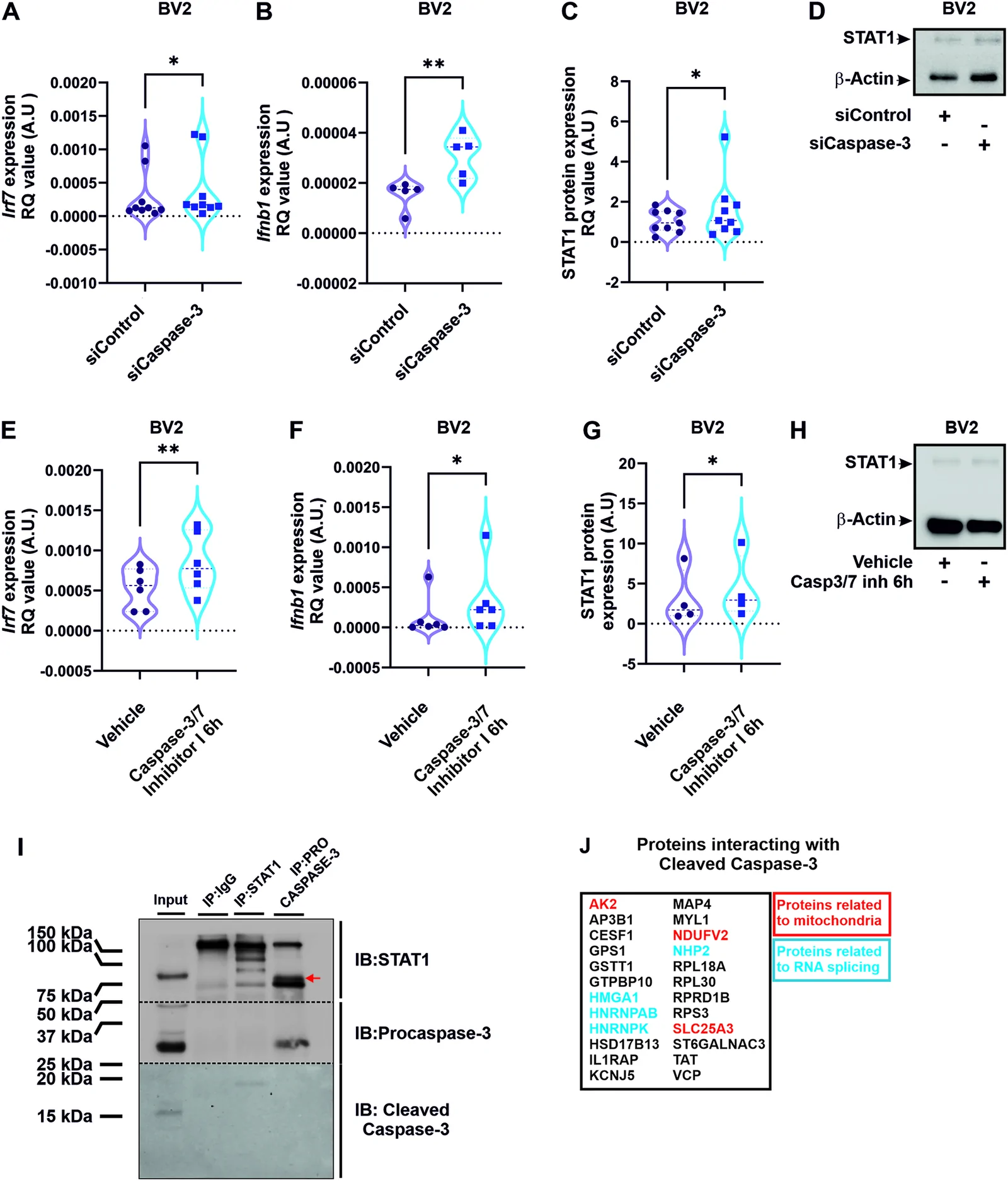
Caspase-3 inhibition in BV2 microglial cells modulates type I IFN signaling and proliferation, recapitulating SN microglia phenotypes. RT-qPCR analysis of mRNA expression levels of *Irf7* (*n* = 9 independent experiments) (**A**), and *Ifnb1* (*n* = 5 independent experiments) (**B**) following siRNA-mediated knockdown of caspase-3 in BV2 cells. Quantification of STAT1 protein levels (*n* = 9 independent experiments) (**C**) and representative immunoblot (**D**) upon caspase-3 silencing. RT-qPCR analysis of *Irf7* (*n* = 6 independent experiments) (**E**) and *Ifnb1* (*n* = 6 independent experiments) (**F**) after 6 h of treatment with caspase-3/7 inhibitor I. Quantification of STAT1 protein (*n* = 4 independent experiments) (**G**) and corresponding immunoblot (**H**) following a 6-h treatment with caspase-3/7 inhibitor I. **I** Co-immunoprecipitation of STAT1 and procaspase-3 under basal conditions in BV2 cells; red arrow indicates lower molecular weight STAT1 species co-precipitated with procaspase-3. **J** Mass spectrometry analysis of proteins bound to cleaved caspase-3 under basal conditions in BV2 cells. Proteins related to mitochondria are highlighted in red; those related to RNA splicing in blue. Statistics: Paired *t*-test (**A**, **B**, **E**, **F**, **G**); paired Wilcoxon matched-pairs signed-rank test (**C**); *p < 0.05, **p < 0.01*, ns* statistically non-significant. See also Supplementary Fig. S5.

To explore the mechanism by which caspase-3 regulates type I IFN responses in vivo, we turned to the BV2 microglial cell line. We validated BV2 microglia as a valid model that could recapitulate key features observed upon caspase-3 loss in vivo. Silencing caspase-3 for 48 h resulted in increased expression of elevated levels of type I IFN-related genes, including Irf7 and *Ifnb1,* and at the protein level, we observed an increase in STAT1 expression, although this was not reflected at the mRNA level (Fig. 5A–D; Fig. S4G). To test if type I IFN effects depend on caspase-3 enzymatic activity, BV2 cells were treated with the Caspase-3/7 Inhibitor I (Fig. S5C, D), inducing the expression of *Irf7* and *Ifnb1* within just 6 h (Fig. 5E, F). At the protein level, STAT1 upregulation was modest but significantly increased following caspase-3/7 inhibition, and again, without corresponding changes at the transcript level (Fig. 5G, H; Fig. S5D). A previous study showed that STAT1 can be a direct substrate of caspase-3 under apoptotic conditions, with cleavage producing a slightly smaller STAT1 isoform [56]. To further investigate this interaction, we performed co-immunoprecipitation experiments and found that caspase-3 and STAT1 can physically interact under basal conditions. Pull-down of procaspase-3 revealed a lower molecular weight STAT1 band (<84 kDa; indicated by a red arrow), and reciprocally, pull-down of STAT1 resulted in detectable levels of cleaved caspase-3 (Fig. 5I). These data suggest that inhibition of caspase-3 may stabilize STAT1 protein levels by preventing its proteolytic processing, thereby facilitating the activation of type I IFN signaling. Analysis using the TRRUST v2 database of transcriptional regulatory interactions (Fig. S5E) revealed STAT1 among the top three transcription factors predicted to modulate genes upregulated once caspase-3 is removed in both genders under saline conditions (Fig. 3A–D).

Given caspase-3′s broad functional role, we investigated its potential interactions with key process regulators. Therefore, we performed immunoprecipitation of cleaved caspase-3 from untreated BV2 microglia, followed by mass spectrometry analysis, using a species-matched IgG as a negative control (Fig. 5J and Fig. S5G). Twenty-four proteins were identified to interact with cleaved caspase-3; some of those proteins were related to mitochondrial metabolism and others to the splicing process. In the case of proteins related to mitochondria, it is noteworthy to mention that the presence of caspase-3 has been shown within this organelle under physiological conditions in various organs [57, 58], including the brain [59]. Such interactions may influence the activity, localization, or stability of these proteins, thereby contributing to changes observed in energy metabolism and splicing regulation observed upon caspase-3 depletion in vivo (Fig. 3C, D and Fig. S3C–J). Notably, one of those proteins, HNRPK, has already been shown to be a target for cleaved caspase-3 under non-apoptotic conditions during the differentiation process in erythrocytes [60]. Given that HNRNPK has been shown to promote Neat1_2 synthesis through regulation of alternative 3′‑end processing [61], our data suggest that modulation of HNRNPK activity downstream of caspase‑3 inhibition could contribute to enhanced paraspeckle formation as we see in Fig. 4F–H.

### Inhibition of caspase-3 in microglia increases intracellular levels of dsRNA

**Fig. 6:**
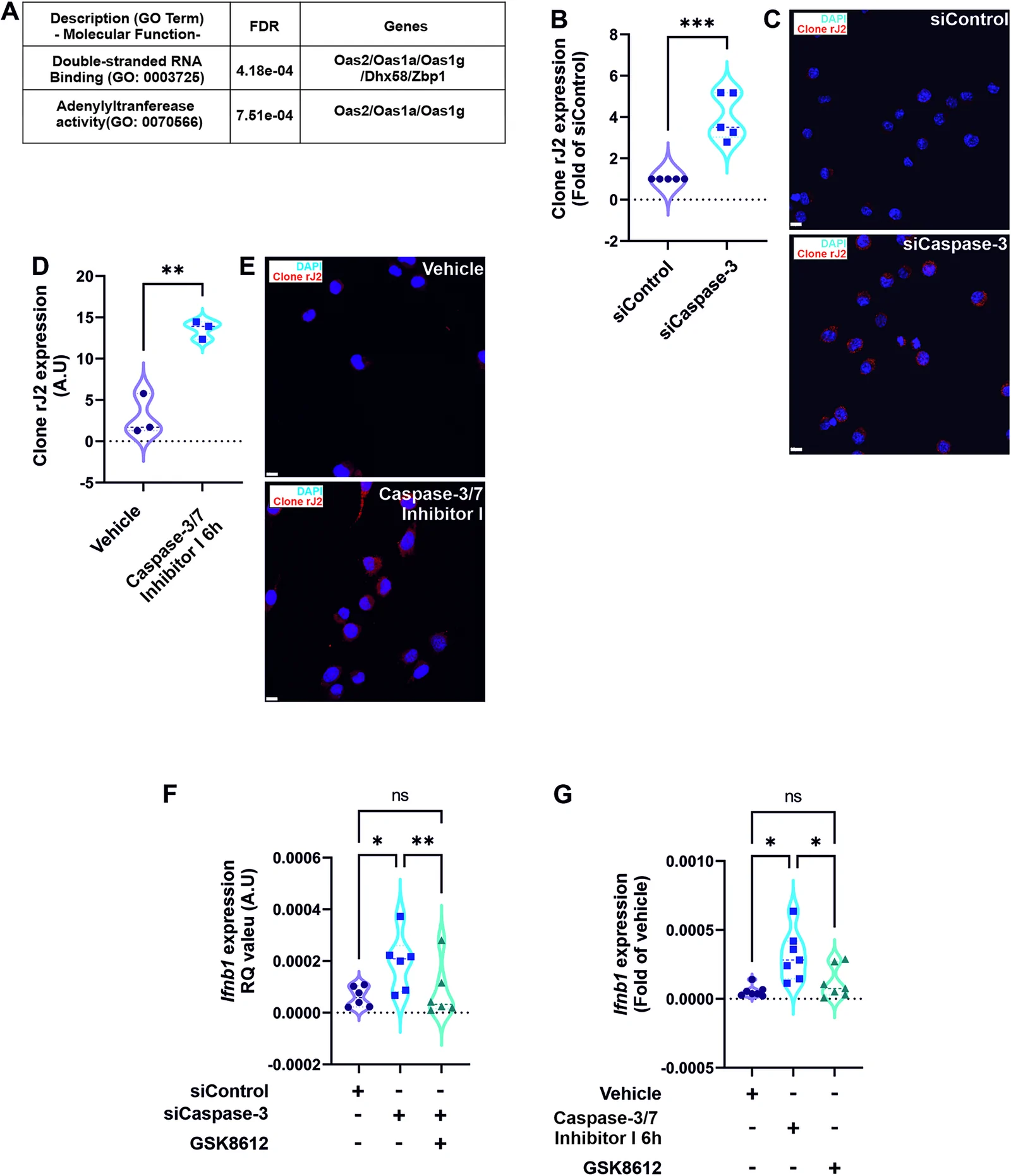
Caspase-3 inhibition in microglia enhances dsRNA accumulation and type I IFN signaling. **A** Table of enriched Gene Ontology (GO) terms related to molecular function in microglia isolated from the substantia nigra (SN) of male *Casp3*^*flox/flox*^*Cx3cr1*^*CreERT2/WT*^ *versus Casp3*^*flox/flox*^ mice under saline conditions. Quantification (**B**) and representative images (**C**) of rJ2 (dsRNA-specific antibody) immunofluorescence (red) in BV2 cells following siRNA-mediated caspase-3 knockdown, scale bar, 25 µm (*n* = 5 independent experiments). Quantification (**D**) and representative images (**E**) of rJ2 immunofluorescence in BV2 cells treated for 6 h with caspase-3/7 inhibitor I (*n* = 3 independent experiments). Scale bar, 10 µm. (F, G) RT-qPCR analysis of *Ifnb1* mRNA expression following TBK1 inhibition with 1 μ GSK8612 (added 6 h prior to harvesting in BV2 cells silenced for caspase-3 (**F**; n = 6) and following a 6-h co-treatment with caspase-3/7 inhibitor I (**G**; *n* = 7). Data in (**B**, **D**) are presented as the mean. Statistics: Ratio paired *t*-test (**B**); paired *t*-test (**D**); one-way ANOVA with Tukey’s *post hoc* test (**F**, **G**). *p < 0.05, **p < 0.01, ***p < 0.001*, ns* statistically on-significant.

Transcriptomic analysis revealed sex-dependent differences in the expression of type I IFN-related genes in microglia in the absence of caspase-3 under saline conditions, with a stronger enrichment observed in males (Fig. 3A–D; Fig. S3A, B). Gene ontology (GO) enrichment analysis, of molecular function terms in males, identified the top two enriched categories as “double-stranded RNA (dsRNA) binding” and “adenylyltransferase activity,” including genes such as *Oas2, Oas1a, Oas1g, Dhx58*, and *Zbp1* (Fig. 6A). These findings suggest that loss of caspase-3 may be associated with the improved sensing of dsRNA, unexpected despite no infection. A potential endogenous source of dsRNA in the absence of viral infection is the accumulation of RNA transcripts with retained introns [37], a phenomenon we observed in Fig. 4 following inhibition of caspase-3/7 activity.

To assess dsRNA build-up in caspase-3-deficient cells, we used J2 immunofluorescence staining for dsRNA >40 bp [59] in BV2 microglia after knocking down caspase-3 (Fig. 6B, C) or treating them for 6 h with the caspase-3/7 inhibitor I (Fig. 6D, E). The results revealed a marked increase in dsRNA staining compared to their controls. To test whether dsRNA contributes to the induction of the type I IFN response, BV2 cells were co-treated with the caspase-3/7 inhibitor or siRNA targeting caspase-3 along with a TBK1 inhibitor (GSK8612, a kinase downstream of dsRNA sensing pathways). In both conditions, the induction of *Ifnb1* expression was completely blocked upon TBK1 inhibitor treatment (Fig. 6F, G). Our results propose that the induction of type I IFN signaling upon caspase-3 inhibition involves three complementary mechanisms: [1] the accumulation of intracellular dsRNA, [2] the stabilization of STAT1 protein levels as shown in Figs. 5D, H, and [3] formation of paraspeckles promoted interferon (IFN) responses by acting as positive feedback for RIG-I signaling [55].

### Loss of caspase-3 causes mitochondrial and energy production dysfunction

**Fig. 7:**
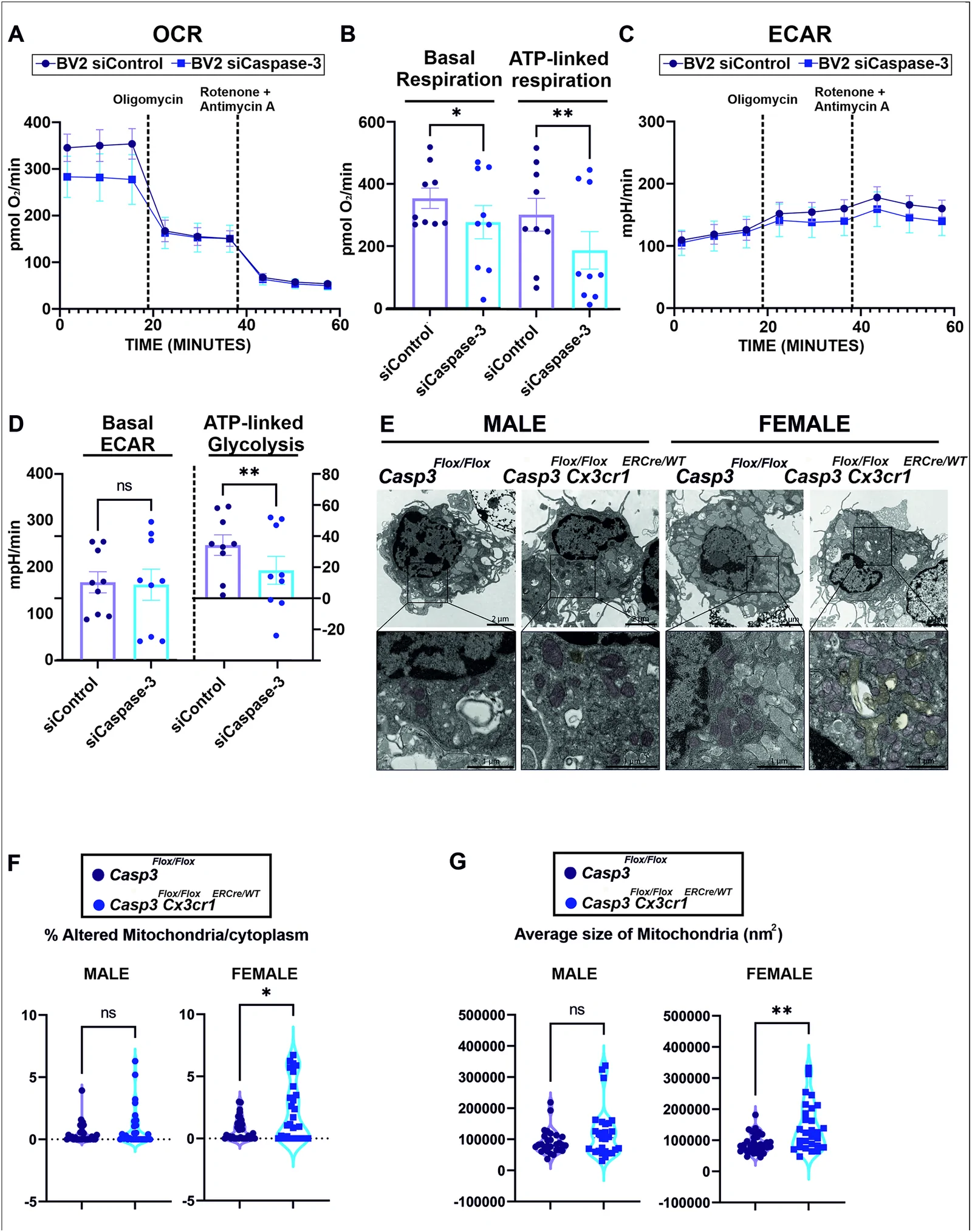
Loss of caspase-3 affects mitochondrial respiration in a sex-dependent manner. **A** Representative Seahorse Bioenergetic Real-Time ATP Rate Assay showing oxygen consumption rate (OCR; pmol O₂/min) in BV2 microglial cells transfected with control siRNA (siControl) or caspase-3-targeting siRNA (siCaspase-3). **B** Quantification of basal respiration and ATP production linked to mitochondrial respiration in BV2 cells treated as in (**A**). Data represent mean ± SEM from three independent experiments with three technical replicates per experiment. **C** Representative Seahorse assay tracing extracellular acidification rate (ECAR; mpH/min) in BV2 cells treated with siControl or siCaspase-3. **D** Quantification of basal ECAR and ATP production linked to glycolysis from (**C**). Data represent mean ± SEM from three independent experiments with three technical replicates per experiment. **E** Representative transmission electron microscopy (TEM) images of primary microglia isolated from male and female *Casp3*^*flox/flox*^ and *Casp3*^*flox/flox*^*Cx3cr1*^*CreERT2/WT*^ mice treated with tamoxifen. Insets highlight examples of healthy (purple) and altered (yellow) mitochondria. Scale bars: 2 μm (main), 1 μm (insets). **F** Quantification of the percentage of cytoplasmic area occupied by altered mitochondria in male and female primary microglia cultures from genotypes as in (**E**). **G** Quantification of average mitochondrial size in cultures as in (**F**). Data in (**F**) and (**G**) represent mean ± SD from three independent experiments, with 10 cells analyzed per treatment group per experiment. Statistics: Paired *t*-test (**B**, **D**); Mann–Whitney U test (**F**, **G**). *p < 0.05, **p < 0.01, *ns* statistically non-significant. See also Supplementary Fig. S6.

In vivo MS analysis under saline conditions revealed that caspase-3-deficiency alters the abundance of proteins involved in energy metabolism and mitochondrial function. To evaluate potential functional effects, we assessed mitochondrial respiration through oxygen consumption rate (OCR) and glycolysis through extracellular acidification rate (ECAR) via Seahorse analysis in BV2 microglia following caspase-3 knockdown. Upon silencing caspase-3, BV2 microglia showed a decrease in basal OCR as well as in ATP production linked to mitochondrial respiration (Fig. 7A, B). However, while no difference was observed at the basal level of ECAR, a decrease in ATP-linked to glycolysis was noted (Fig. 7C, D). Notably, our in vitro IP-proteomic data in Fig. 5J showed that cleaved caspase-3 physically interacts with several proteins involved in mitochondrial metabolism, further supporting its role in regulating cellular energy homeostasis.

In vivo mass spectrometry revealed sex-dependent changes in energy metabolism proteins in caspase-3-deficient microglia, with females showing a stronger response (Fig. 3E). To assess if molecular changes correlated with mitochondrial structural alterations, we performed transmission electron microscopy (TEM) on gender-segregated primary microglia from tamoxifen-treated *Casp3*^flox/flox^ and *Casp3*^flox/flox^*Cx3cr1*^CreERT2/WT^ mice (Fig. S6A, B). Microglia from female *Casp3*^flox/flox^*Cx3cr1*^CreERT2/WT^ mice showed a significant increase in the proportion of cytoplasmic area occupied by altered mitochondria compared to microglia from female control *Casp3*^flox/flox^*Cx3cr1*^CreERT2/WT^ mice (Fig. 7E, F). Altered mitochondria were defined by disorganized cristae and/or disrupted inner or outer membranes, features associated with mitochondrial dysfunction and impaired ATP production. Additionally, female *Casp3*^flox/flox^*Cx3cr1*^CreERT2/WT^ microglia displayed increased mitochondrial size, consistent with the presence of swollen mitochondria (a hallmark of mitochondrial stress) (Fig. 7G). Other factors such as area of the cell, area of the cytoplasm, area of the nucleus, total number of mitochondria, and the % of cytoplasm occupied by non-altered mitochondria were not affected by loss of caspase-3, either in males or females (Fig. S6C–G).

## Discussion

In this study, we aimed to investigate the role of caspase-3 in the regulation of microglial immune responses in the context of PD. Our initial hypothesis, based on previous work from our group, was that caspase-3 activation under inflammatory conditions modulates the immune response independently of its classical role in apoptosis. Overall, our data support a unified model in which disruption of basal caspase-3 activity impairs RNA splicing fidelity, promotes accumulation of endogenous dsRNA, and activates a type I interferon response in microglia. We observed only limited activation of caspase‑3 in microglia following MPTP treatment. In a small cohort of PD patients, approximately 20–40% of microglia exhibited cytosolic caspase‑3 cleavage. The discrepancy between mouse and human data may reflect the chronic nature and greater severity of neurodegeneration in PD patients compared with the acute MPTP model. Importantly, we cannot confidently determine the caspase‑3 status of the remaining microglial population lacking detectable cleavage, although these findings suggest a non‑homogeneous microglial population with respect to caspase‑3 activity. Surprisingly, our findings reveal a previously unrecognized role for basal caspase-3 activity in maintaining microglial homeostasis and shaping the nature of the inflammatory response. Specifically, we demonstrate that the loss of caspase-3 disrupts several fundamental microglial processes, including type I IFN signaling, mitochondrial dysfunction, and RNA splicing, all of which are biological features known to be altered in PD, as observed both in animal models and in patients with PD [19–23, 62]. Alternative splicing has been reported in genes directly implicated in PD pathogenesis, including *PARK2, SNCAIP, LRRK2, SNCA, SRRM2, MAPT*, and *DJ-1* [39]. In addition to generating pathogenic protein isoforms, dysregulated RNA splicing can lead to the formation and accumulation of aberrant double-stranded RNA (dsRNA) intermediates [40, 41], contributing to neuroinflammation and neurodegeneration in PD [42]. Our mass spectrometry analysis reveals substantial alterations in the abundance of splicing-associated proteins in caspase-3-deficient microglia, including several RNA-binding proteins (e.g., HNRNPs, SNRNPs, and SF3 family members). Also, short inhibition of caspase-3/7 activity provoked significant changes in differential exon usage, with an enrichment of transcripts containing retained introns, indicative of impaired splicing fidelity that could lead to an increase in intracellular dsRNA in different manners [63–65]. Similarly, short-term inhibition of caspase-3/7 activity in primary microglia altered the expression of transcripts known to modulate splicing regulation, such as the long non-coding RNA Neat1 and particularly its long isoform Neat1_2, which is responsible for paraspeckle formation. It has been shown that stimulation by Poly I:C (a dsRNA-mimicking immunostimulant) can induce Neat1_2 expression [53], acting as a positive regulator of innate immune signaling, including the type I IFN response [55, 66]. Supporting the relevance of this finding, a recent study conducted in MPTP-treated mice [67] reported an increase in Neat1_2 early after MPTP injection. Altogether, the increase in dsRNA, enhanced paraspeckle formation, and stabilization of STAT1 upon inhibition of basal caspase‑3 activity provide mechanistic insight into how loss of caspase‑3 activity may promote a type I IFN response.

Noteworthy, a recent study by the Molofsky lab [68] demonstrated that IFN-I-responsive microglia actively engulf whole neurons during cortical development, and that disruption of this process results in excitatory/inhibitory imbalance and tactile hypersensitivity. Interestingly, our findings may align with this work, as caspase-3/7 inhibition in microglia induces a type I IFN response, and it has also been shown by others to promote phagocytosis of neuronal elements [16, 17].

Furthermore, integrated metabolic flux analysis and proteomic profiling reveal that the loss of caspase-3 results in a significant disruption of mitochondrial function, as evidenced by decreased oxygen consumption and reduced ATP production. Our immunoprecipitation-mass spectrometry data offers a direct molecular link between caspase-3 and the coordination of mitochondrial and splicing machinery, further implicating its basal activity in maintaining microglial homeostasis. Significantly, mitochondrial dysfunction and spliceosome dysregulation are not totally independent processes. The splicing process is highly ATP-dependent, and it has been shown that energy deficits can severely impair splicing efficiency [44]. Conversely, aberrant splicing can alter the expression of proteins essential for mitochondrial respiration, potentially exacerbating mitochondrial defects [39].

Sex-dependent effects emerged as a striking and consistent feature of caspase-3-deficiency in our study. Female microglia showed greater metabolic susceptibility, with decreased expression of proteins related to energy metabolism as shown by mass spectrometry in vivo and more extensive mitochondrial damage observed by electron microscopy in gender-segregated primary microglia in vitro, suggesting that cell signaling processes such as skewed X-chromosome inactivation [69–71] play a major role in these differences. The sex-specific engagement of caspase-3 in maintaining mitochondrial integrity raises the interesting possibility that female microglia are more reliant on caspase-3-mediated regulatory networks. Also, under physiological conditions, females have a more robust mitochondrial respiratory capacity and ATP production than males; however, during pathological conditions such as PD, females tend to present with more dysfunctional lysosomal capacity compared to males, which could lead to accumulation of altered mitochondria [49, 50] that may help explain differences observed in our gender-segregated primary microglia data.

Previous studies under cell death conditions have shown that females preferentially engage caspase-dependent cell death pathways, while males tend to favor caspase-independent mechanisms [45, 46, 48]. Further, our transcriptomic data did not reveal sex differences in caspase-3 mRNA expression, nor in key upstream activators or inhibitors of caspase-3 (e.g., XIAP). This implies that the functional divergence arises not from differential transcription but likely from post-translational regulation, subcellular localization, or differences in protein–protein interactions. One post-translational modification of caspase-3 is S-nitrosylation at a critical cysteine residue, which inhibits its processing and activation [15, 72]. Notably, in vivo MPTP treatment has been shown to promote an oxidative environment in the substantia nigra that facilitates the S-nitrosylation of other proteins, such as Parkin [73], opening the possibility that caspase-3 could also be affected by S-nitrosylation in microglia.

A limitation of this study is the small number of biological replicates in our omics datasets. However, this study was intended to be an exploratory investigation aimed at identifying signaling pathways affected by the loss of caspase-3, which would then be subsequently validated by more robust and mechanistically relevant techniques.

Taken together, our findings advance the understanding of caspase-3 as a regulator of microglial RNA splicing and bioenergetics. They also highlight the need to dissect the post-translational and interactome-level regulation of caspase-3, particularly in female microglia. Future studies will be critical to uncover the mechanistic basis for these sex-specific metabolic phenotypes.

## Material and methods

### Mice and MPTP treatment

Both male and female mice were employed in this study. Mice were housed in specific pathogen-free conditions under standard 12-h light/dark cycle conditions in rooms equipped with controls for temperature (21 ± 1.5 °C) and humidity (50 ± 10%). *Casp3*^*flox/flox*^ C57BL6/J mice with the Casp3 allele floxed at exon 2 and C57BL/6/J mice containing a Cre recombinase under the control of Cx3cr1 promoter and enhancer elements (Jackson Laboratories, B6.129P2(Cg)-Cx3cr1tm2.1(cre/ERT)), were crossed to generate *Casp3*^*flox/flox*^*Cx3cr1*^*CreER2/WT*^ (microglial Casp3 KO mice) and Casp3^flox/flox^Cx3cr1^WT/WT^ (control mice). Deletion was induced upon daily tamoxifen treatment (2 mg of tamoxifen diluted in 100 μl of 5% ethanol in oil) for five consecutive days starting at 10–12 weeks of age. All mice (*Casp3*^*flox/flox*^*Cx3cr1*^*CreER2/WT*^ and *Casp3*^*flox/flox*^*Cx3cr1*^*WT/WT*^) were injected with tamoxifen. 19 days after the last injection with tamoxifen, mice were injected four times intraperitoneally in a 2 h interval with either saline or MPTP (16 mg/Kg diluted in saline) [9]. Animals were sacrificed four days after the final injection. During the injection period, cages containing both saline- and MPTP-treated mice were placed on heat blankets. Additionally, all animals received intracutaneous saline injections to prevent dehydration. Data was analyzed first, separated by sex to check for any significant sex differences before combining.

Animals were assigned to experimental groups based on genotype rather than formal randomization. This strategy was used to control for genetic and developmental variability. Investigators were blinded to treatment conditions during data analysis where feasible.

### Cell line and siRNA transfection

The murine microglial BV2 (female) cell line was cultured as described in [15]. Briefly, the cells were maintained in 10% fetal bovine serum (FBS) in DMEM and, during the experiments, supplemented with penicillin/streptomycin (100 U/ml and 100 μg/ml respectively). Transfection of BV2 cells was carried out using the Magnetofection technique for 48 h prior to the experiments (OzBiosciences) following the manufacturer’s instructions as described in [74] with minor modifications.

### Patient material

Human brain tissue was obtained from 3 males (PD subjects 1, 2, and 3) and 1 female (Control subject). The region investigated was the *substantia nigra*. All sections were stained with antibodies against cleaved caspase-3 and IBA1. They were microscopically reviewed for verification of pathology. Before the investigation, the entire collection of brain sections was subjected to a neuropathological whole-brain analysis for clinical diagnostic purposes, according to routine procedures at the University Hospital Clinic (Barcelona)/IDIBAPS. The project procedures involving human brain tissue were approved by the Regional Ethical Review Board, reference number (0025-N-21).

### Primary microglia cell cultures

Primary postnatal mouse microglial cells were prepared from P1–4 experimental and control mouse brains following the described protocol [75]. BALB/c strain mice were used to generate gender mixed primary microglia cell cultures for RNA-seq experiments.

Postnatal P1–4 mice were sacrificed, and brains were carefully dissected, removing all the meninges and cerebellum, and the cortices were washed in ice-cold Ca^2+^- and Mg^2+^-free Hanks’ buffered salt solution (HBSS; Biowest). Later on, brains were minced and resuspended in ice-cold HBSS. After being washed, tissues were incubated for 15 min at 37 °C in HBSS containing 0.1% trypsin and DNase 0.05% and resuspended in DMEM medium containing 10% FBS and 1% P/S (DMEM, high glucose, Glutamax^TM^, Gibco). Cells isolated from 1 brain were cultivated in a T25 flask, whereas a pool of cells from 4 to 5 brains was cultivated in a T75 flask. Medium was replaced completely after 1 day of seeding and was supplemented with GM-CSF (5 ng/ml) 7 days after seeding.

Deletion was induced upon 4-OH-tamoxifen treatment for 96 h before harvesting microglia. Microglial cells were harvested from confluent astrocyte monolayers, 10–12 days after the initial seeding, by tapping the side of the culture flask. These microglial cells found in the medium were plated into 24-well dishes. Experiments were performed 24 h after the final plating.

### RT-qPCR analysis

Total RNA was extracted using the TRI Reagent^@^ reagent (Sigma-Aldrich) following the manufacturer’s instructions. Using the RevertAid First Strand cDNA Synthesis Kit (Thermo Scientific, Spain), 1 μg of the total RNA was transformed into cDNA. qPCR was performed using the SensiFast SYBR no-ROX kit (Meridian) and Taqman Universal PCR Master Mix (Thermo Fisher Scientific). Results were calculated using the delta Ct method and represented as absolute values with arbitrary units. *Actb* gene was used as housekeeping in SYBR-based RT-qPCR, and *Pgk1* was used as housekeeping in Taqman-based RT-qPCR.

### Isolation of adult microglia

Adult mice were deeply anesthetized and transcardially perfused with ice-cold saline to remove circulating blood cells. Brains were rapidly extracted, and the substantia nigra (SN) was dissected. For microarray assays, one SN was collected per animal, while for mass spectrometry analyses, two SNs from two animals were pooled. Dissected tissue was minced in Hanks’ Balanced Salt Solution (HBSS) using a sterile scalpel and then mechanically dissociated by passing the homogenate through a 20-gauge needle 12 times. The resulting suspension was passed through a 70 μm cell strainer (Falcon) to remove tissue debris. The filtrate was then centrifuged at 160 × *g* for 10 min at 4 °C in a swinging bucket centrifuge with brake. The pellet was resuspended in 3 mL of 30% Percoll (GE Healthcare) diluted in HBSS (prepared from a stock 90% Percoll solution made in HBSS the day prior). To establish a discontinuous gradient, 2 mL of HBSS was gently layered on top of the cell suspension. Samples were centrifuged at 800 × *g* for 15 min at 4 °C without brake. Following centrifugation, the myelin-containing upper layer and supernatant were carefully removed using a glass pipette. The cell pellet was washed once with 4 mL of PBS and centrifuged again at 800 × *g* for 10 min at 4 °C with brake. After removing the supernatant, microglia were isolated using CD11b MicroBeads and MACS columns (Miltenyi Biotec) according to the manufacturer’s protocol with minor modifications. All steps were performed at 4 °C to preserve protein integrity.

### Electron microscopy preparation of samples and analysis

Primary male and female microglia, cultured in T25 flasks and treated with tamoxifen for 96 h, from *Casp3*^*flox/flox*^*Cx3cr1*^*CreER2/WT*^ and *Casp3*^*flox/flox*^ mice were harvested and washed twice with sterile PBS to remove medium residues for ultrastructural analysis of their mitochondria. After removing the PBS, the cell pellet was fixed with 1.6% glutaraldehyde diluted in cacodylate buffer for 15 min at RT. The sample was then centrifuged again at 1000 × *g* for 2 min and washed three times with cacodylate buffer, in which it was finally resuspended. Then samples were post-fixed for 1 h at 4 °C in 1% OsO4 in the same buffer. After three washing steps (20 min each), the samples were immersed in 2% uranyl acetate, dehydrated through a gradient acetone series (50%, 70%, 90% and 100%), and embedded in Spurr resin. Blocks were obtained by polymerization at 70 °C for 8 h. Thin sections were cut with a diamond knife using an ultramicrotome (Leica UC7) and examined with a transmission electron microscope (Zeiss Libra 120) operating at 80 kV. The electron microscopy images were taken at the same magnification (3969x) with an EMCCD camera (TRS 2k x 2k).

Gender-segregated primary microglia were analyzed for various ultrastructural parameters pertaining to mitochondria. First, the cell area and nucleus were measured to obtain the area of the cytoplasm. Mitochondria were positively identified by their double membrane, electron-dense appearance, and the presence of cristae [76]. Altered mitochondria are defined by the presence of a degradation of the inner or outer membrane, swollen cristae that appear electron-lucent, or overall degradation of the mitochondria [77]. Elongated mitochondria are positively identified if they measure more than 1000 nm in length [78]. Non-altered mitochondria present none of the parameters previously mentioned (no visible sign of ultrastructural alterations and measure less than 1000 nm in length). Each mitochondrion was outlined, and its area was calculated using ImageJ. The overall percentage of space that each category of mitochondria (non-altered, elongated, altered) occupies within the cytoplasm was calculated. The number of mitochondria was calculated as well as the relative percentage of each category of mitochondria (e.g., % of altered mitochondria over all mitochondria). The average size of all mitochondria within one cytoplasm was also measured.

### LC-MS/MS with an Easy-nLC 1000 HPLC system (Thermo Fisher) coupled to a Q Exactive Plus Orbitrap mass spectrometer

Microglia isolated from the substantia nigra (SN) using CD11b magnetic columns (Miltenyi Biotec) were centrifuged at 2500 × *g* for 5 min at 4 °C, and the supernatant was discarded. Cell pellets were resuspended and snap-frozen in 20 μL of lysis buffer containing 0.2% n-dodecyl β-D-maltoside (DDM; Thermo Scientific), 100 mM triethylammonium bicarbonate (TEAB; Supelco), 1% protease inhibitor cocktail (Sigma-Aldrich), 1% phosphatase inhibitor cocktails II and III (Sigma-Aldrich), and 0.1% benzonase nuclease (Thermo Scientific).

Afterward, analysis of digested peptides was performed by LC-MS/MS with an Easy-nLC 1000 HPLC system (Thermo Fisher) coupled to a Q Exactive Plus Orbitrap mass spectrometer (Thermo Fisher). Chromatographic separation was performed with a C18 HPLC precolumn (75 μm × 2 cm) and Easy Spray HPLC column (75 μm × 25 cm, 2 μm, 100 Å). Gradient elution was performed with a binary solvent system consisting of (A) 0.1% aqueous formic acid and (B) 0.1% formic acid in acetonitrile (CH3CN), with a 120 min gradient from 5% to 40% B at a flow rate of 250 nl/min. An MS survey scan was obtained using a top 15 method with a 100–2000 m/z mass range. The MS/MS Spectra Acquisition was obtained using Higher-energy Collisional Dissociation (HCD). An isolation mass window of 2 m/z was used for the precursor ion selection, and a normalized collision energy of 27% was used for fragmentation. A 10 s dynamic exclusion was applied.

### LC-MS/MS with a nanoElute II LC system coupled to a timsTOF SCP mass spectrometer with an electrospray source (Bruker Daltonics)

For samples immunoprecipitated with anti–cleaved caspase-3 and IgG isotype control, bound proteins were eluted after the final wash by adding 50–100 μL of acidic elution buffer (200 mM glycine, pH 2.5), followed by pipetting up and down for 30–60 s at room temperature. Eluates were transferred to fresh tubes and immediately neutralized with 5–10 μL of 1 M Tris-HCl, pH 10.4. The elution step was repeated once to maximize recovery.

Proteins were reduced and cysteine residues blocked with 50 mM tris-(2-carboxyethyl) phosphine (TCEP, AB Sciex) and chloroacetamide (CAA, Sigma-Aldrich, St. Louis, MO, USA) for 30 min at 37 °C with shaking. Proteolysis was carried out with trypsin (Promega, Fitchburg, WI, USA) at 1:20 (enzyme to substrate) at 37 °C overnight with agitation following the FASP standard protocol using Millipore Microcon 30 K filters (Merck, Darmstadt, Germany) [79]. The resulting peptide samples were collected by centrifugation, dried in a vacuum evaporator, and desalted using a Stage-tip (Omix C18 tips, Agilent). Sample injection volumes were normalized to ensure equal protein loading across samples. Analysis of digested peptides was performed by LC-MS/MS with a nanoElute II LC system coupled to a timsTOF SCP mass spectrometer with an electrospray source (Bruker Daltonics). Chromatographic separations were performed on a C18 HPLC column (Aurora 25 cm and 75 µm ID, IonOpticks, Australia) kept at 50 °C. Gradient elution was performed with a binary system consisting of (A) 0.1% aqueous formic acid and (B) 0.1% formic acid in acetonitrile during 60 min from 5 to 37% gradient of B with a flow rate of 250 nl/min. Mass spectrometric analysis was performed in a data dependent acquisition parallel accumulation serial fragmentation (DDA-PASEF) mode with 100–1700 m/z mass range, an ion mobility ranges from 0.70 to 1.30 V s cm^−2^, capillary voltage set to 1500 V, an accumulation and ramp time at 100 ms and the collision energy as a linear ramp from 20 eV at 1/K0 = 0.6 V s cm^−2^ to 59 eV at 1/K0 = 1.6 V s cm^−2^.

### Microarray for in vivo transcriptomic analysis of microglia

Microglia isolated from the substantia nigra (SN) using CD11b magnetic columns (Miltenyi Biotec), centrifuged at 2500 × *g* for 5 min at 4 °C, were resuspended using TRI Reagent® and RNA was isolated following the manufacturer´s protocol. RNA was cleaned of salts and solvent contaminants by a series of ethanol washes previous to quality analysis.

Microarray was performed in the Genomic facility of the IBiS. The RNA quality was analyzed using a 4200 TapeStation system (Agilent). All samples were amplified and labeled using the GeneChip® 3′ IVT pico Kit (Thermo Fisher Scientific, Inc.). Amplification was performed with 1–2 ng of total RNA input following procedures described in the 3′ IVT pico Kit user manual. The amplified cDNA was quantified, fragmented, and labeled in preparation for hybridization to GeneChip® Clariom S Mouse Array (Thermo Fisher Scientific, Inc.) using 5 μg of double-stranded cDNA product and following protocols outlined in the user manual. Washing, staining (GeneChip® Fluidics Station 450, Thermo Fisher Scientific, Inc.), and scanning (GeneChip® Scanner 3000, Thermo Fisher Scientific, Inc.) were performed following protocols outlined in the user manual for cartridge arrays.

### Tissue preparation and sectioning

Male and female mice were deeply anesthetized and transcardially perfused with ice-cold 0.9% sodium chloride (NaCl). Brains were rapidly dissected, post-fixed in 4% paraformaldehyde (PFA) at 4 °C, and cryoprotected in 30% sucrose in phosphate-buffered saline (PBS) until saturated. Brains were then embedded and frozen, and coronal sections (30 μm thickness) were cut using a cryostat (–20 °C) and mounted onto glass slides. Sections were stored at –20 °C until further processing.

### Immunohistochemistry

Slides containing coronal brain sections were subjected to antigen retrieval in 10 mM citrate buffer (pH 6.0) using a 2100 Antigen Retriever (Aptum Biologics), following the manufacturer’s instructions. Sections were then incubated in 3% hydrogen peroxide (H₂O₂) and 10% methanol in phosphate-buffered saline (PBS, pH 7.4) for 20 min to quench endogenous peroxidase activity. Following this, tissues were permeabilized and blocked in PBS containing 0.2% Triton X-100 (PBS-T) and 5% normal horse serum (NGS) for 1 h at room temperature. After blocking, sections were incubated overnight at 4 °C with rabbit anti-tyrosine hydroxylase (TH) primary antibody (1:500, Sigma-Aldrich) diluted in blocking solution. The following day, sections were incubated for 1 h with biotinylated horse anti-rabbit IgG secondary antibody (1:200, Vector Laboratories, BA-1100), followed by a 1 h incubation with the avidin–biotin complex (ABC; Vector Laboratories, PK-6100). Signal was visualized using the DAB Substrate Kit (Vector Laboratories, SK-4100), and the reaction was stopped by rinsing in PBS. Sections were dehydrated through a graded ethanol series, cleared in xylene, and mounted using DPX mounting medium (Sigma-Aldrich).

### Quantification of tyrosine hydroxylase (TH) immunoreactivity in striatum

Quantification of TH immunoreactivity in the striatum was performed using Fiji/ImageJ software (NIH, Bethesda, MD, USA). Images of TH-stained striatal sections were acquired using an OLYMPUS BX61 microscope (Olympus, Tokyo, Japan) under identical exposure settings for all conditions. Regions of interest (ROIs) encompassing the striatum were manually delineated, and the mean signal intensity within each ROI was measured. Background signal was subtracted from each measurement to correct for non-specific staining. Optical density was calculated based on a calibrated grayscale and averaged across multiple sections per animal to obtain representative values for each experimental condition.

### Stereological quantification of dopaminergic neurons in the substantia nigra

Stereological analysis of tyrosine hydroxylase (TH)-positive neurons in the substantia nigra (SN) was performed to estimate the total number of dopaminergic neurons. Immunostained brain sections (30 μm thickness) were analyzed using the newCAST stereology software (Visiopharm, Hørsholm, Denmark). A defined region of interest (ROI) spanning 300 μm along the anterior–posterior axis of the SN was assessed. For each animal, five coronal sections were systematically sampled at 100 μm intervals, starting from a random point within the ROI.

Neuron counting was conducted using the optical fractionator method, as described in [18]. Within each section, an unbiased counting frame (40 μm × 25 μm; area = 1000 μm²) was applied using a 20× objective. Sampling was performed at fixed intervals of 150 μm (x-axis) and 200 μm (y-axis), yielding an area sampling fraction of 0.033. The full z-depth of each section was analyzed, resulting in a section thickness sampling fraction of 1. A section sampling fraction of 0.30 was calculated based on 30 μm sections collected every 100 μm. The total number of TH-positive neurons in the SN was estimated by multiplying the raw counts by the inverse of both the area and section sampling fractions.

### Immunofluorescence for mouse material

For double immunofluorescence labeling of IBA1 and cleaved caspase-3, brain sections were first subjected to antigen retrieval (10 mM citrate buffer pH 6.0 using a 2100 Antigen Retriever antigen following the manufacturer´s instructions), followed by permeabilization and blocking in PBS containing 0.3% Triton X-100 and 10% (v/v) normal donkey serum (NDS) for 1 h at room temperature. The tissue was then incubated overnight at 4 °C with the primary antibodies against cleaved caspase-3 (1:300, Cell Signalling) and IBA1 (1:500, Abcam). The next day, sections were washed three times in PBS and incubated for 1 h at room temperature with species-specific Alexa Fluor–conjugated secondary antibodies (1:500, Invitrogen). Nuclei were counterstained with DAPI (1 µg/mL, Sigma-Aldrich). Sections were mounted using ProLong™ Diamond Antifade Mountant (Invitrogen) and imaged using a Leica Stellaris 8 FALCON inverted confocal laser-scanning microscope equipped with a 20× objective.

### Immunofluorescence staining of BV2 cells

Immunofluorescence staining was performed in BV2 microglial cells cultured on glass coverslips in 24-well plates to detect double-stranded RNA (dsRNA). Cells were washed twice with PBS and fixed with 4% paraformaldehyde for 15 min at room temperature. After fixation, cells were washed three times with PBS (5 min each) and permeabilized with ice-cold methanol for 10 min at −20 °C. Following permeabilization, cells were blocked for 1 h at RT in PBS containing 0.3% Triton X-100 and 5% normal donkey serum (NDS). Cells were then incubated overnight at 4 °C with mouse anti-dsRNA antibody (clone J2, Sigma-Aldrich; 1:60), or with rabbit anti-SPFQ (Abcam; 1:250) diluted in blocking solution. The following day, cells were washed three times in PBS (5 min each) and incubated for 1 h at room temperature with fluorophore-conjugated secondary antibodies (1:500, Invitrogen) diluted in blocking buffer. After 3 more PBS washes, nuclei were counterstained with DAPI (1:1000 dilution of a 1 mg/mL stock solution). Coverslips were mounted with ProLong™ Diamond Antifade Mountant (Thermo Fisher Scientific).

Images were acquired using a Leica Stellaris 8 FALCON inverted confocal microscope with a 20× objective. Five fields per treatment condition were captured under consistent imaging parameters (laser intensity, exposure, and photomultiplier settings), typically on the same day. Image analysis was performed using Fiji/ImageJ (NIH, Bethesda, MD, USA). dsRNA-positive areas were quantified by generating binary masks from maximum projection images using identical thresholds across all conditions. Percentage of dsRNA signal localized was normalized to cell number.

For paraspeckle analysis, images were acquired using identical acquisition settings across experimental conditions. Z-stacks encompassing the full nuclear volume were collected, and maximum-intensity projections were used for analysis. Quantification was performed in FIJI/ImageJ using a standardized workflow. Nuclei were segmented based on DAPI signal, and SFPQ-positive nuclear bodies were detected using intensity-based thresholding and size filtering to exclude diffuse nuclear signal.

### Immunobloting

Whole-cell extracts were prepared in RIPA buffer (Sigma-Aldrich) and incubated on ice for 30 min with vortexing every 10 min. Lysates were sonicated on ice (3 cycles of 10 pulses at 80% amplitude, 0.5 duty cycle) using a UP50H ultrasonic processor (Hielscher Ultrasonics). After centrifugation at 15,000 × *g* for 15 min at 4 °C, supernatants were collected, and protein concentration was determined using the BCA assay (Thermo Scientific). Samples were boiled for 5 min in Laemmli buffer, and 30 μg of total protein per sample was resolved on 12% or 15% SDS–PAGE gels at 150 V, followed by transfer to 0.2 μm nitrocellulose membranes for 2 h at 200 mA. Membranes were blocked overnight at 4 °C in blocking buffer (50 mM Tris-HCl, pH 7.5, 500 mM NaCl, 5% non-fat milk), then incubated with primary antibodies overnight at 4 °C in blocking buffer supplemented with 1% BSA. Membranes were washed in PBS and incubated with HRP-conjugated secondary antibodies (Vector Laboratories) for 1 h at room temperature. Signal was detected using enhanced chemiluminescence (ECL) according to the manufacturer’s instructions. A list of primary antibodies is provided in the Key Resources Table. β-actin (Sigma-Aldrich) was used as a loading control.

### Immunoprecipitation

Protein–protein interactions were assessed by immunoprecipitation (IP) in BV2 cells, following the method described previously [10]. Cells were lysed post-treatment in IP buffer (20 mM Tris-HCl, pH 7.5, 140 mM NaCl, 1% Triton X-100, 2 mM EDTA, 1 μM p-amidinophenylmethanesulfonyl fluoride hydrochloride, 50 mM NaF, and 10% glycerol) and homogenized by sonication (1 pulse, 30 cycles at 80% amplitude, 0.5 duty cycle) using a UP50H ultrasonic processor (Hielscher Ultrasonics). Lysates were cleared by centrifugation, and 2 mg of total protein per sample was used for IP.

Supernatants were pre-cleared with protein A/G magnetic beads (Thermo Scientific) for 1 h at 4 °C on an end-over-end rotator. Cleared lysates were incubated overnight at 4 °C with 150 ng of anti-caspase-3 (Cell Signaling), anti-STAT1 (Cell Signaling), or control rabbit IgG (R&D Systems) in a final volume of 150 μL. The next day, protein–antibody complexes were captured by incubation with protein A/G magnetic beads for 1 h at 4 °C on a rotator. Beads were washed four times in wash buffer (50 mM Tris-HCl, pH 7.5, 0.1% SDS, 1% Igepal, 62.5 mM NaCl) at 4 °C, and bound proteins were eluted in denaturing sample buffer for immunoblot analysis.

### Measurements of OCR and ECAR

OCR and ECAR were measured using a Seahorse XF-96 Extracellular Flux Analyzer (Agilent Technologies) following the manufacturer’s instructions and as previously described [80]. BV2 cells transfected with control or caspase-3 siRNA for 48 h were detached by pipetting and seeded at 25,000 cells per well in Seahorse XF-96 cell culture plates. Cells were briefly centrifuged at 300 × *g* for 2 min to increase attachment. Cells were incubated in Seahorse XF assay medium (25 mM glucose, 2 mM glutamine, 1 μΜ sodium pyruvate) at 37 °C in a non-CO₂ incubator for 1 h prior to analysis.

The sensor cartridge was hydrated overnight in XF Calibrant (pH 7.4) at 37 °C in a regular incubator. After cell detachment and counting, cells were resuspended in assay medium at 5 × 10⁵ cells/ml, and 50 μl of cell suspension was added per well. Plates were centrifuged at 300 × *g* for 5 min to facilitate attachment and incubated for an additional hour at 37 °C without CO₂. OCR and ECAR were measured under basal conditions and following sequential injection of oligomycin (2 μM), rotenone (1 μM) plus antimycin A (1 μM).

### RNA-seq

Reads were de-multiplexed and trimmed using FASTQ Generation based on bcl2fastq. Raw samples were concatenated, and quality control (fastQC) was performed. Then, reads were mapped to the murine genome assembly (GRCm39) using HISAT2 [81] using default parameters but specifying --rna-strandness RF. Genome Coverage was calculated using ‘bamCoverage’ from deepTools [82] using default parameters but dividing the genome into 1 bp bins and normalizing mapped reads by CPM. The BedGraph files from each time point generated with bamCoverage were strand-specific. All of this procedure was performed at Cabimer’s computational cluster by customized scripts.

Differentially expressed genes between samples were analyzed using edgeR [83]; gene positions were obtained from the GTF file from GENCODE (M36, GRCm39). We considered differentially expressed genes when log2FC > 1 (upregulated) or Log2FC < −1 (downregulated) with FDR < 0.05. Volcano plots were generated using an R custom script, plotting Log2FC (x-axis) and Log10 of the FDR (y-axis). Alternative splicing analyses comparing samples treated with the caspase-3/7 inhibitor (INH) or vehicle were performed using DEXSeq [84]. For differential exon usage (DEU) analyses, the same GTF file from GENCODE was used. We considered significant DEU events when log2FC > 1 (upregulated exons) or Log2FC < −1 (downregulated exons) with *p*-value < 0.05. Volcano plots were generated as explained above.

### Dataset analyses

Experimental conditions were defined across biological sex (male, female), treatment (saline, MPTP), and genotype (*Casp3*^*flox/flox*^ and *Casp3*^*flox/flox*^*Cx3cr1*^*CreERT2/WT*^). For MS/MS datasets, proteins were quantified by spectrometry-based label-free identification. Mass spectrometry data analysis was performed through MaxQuant software against the Uniprot Mus musculus database and filtered to a false discovery rate (FDR) below 1%. Proteins were considered present in one condition if two or more unique peptides were detected in both samples. Proteins identified as potential contaminants and reverse hits were excluded. For microarray gene expression datasets, differentially expressed genes were filtered using an adjusted *p-value* threshold of ≤0.05 and absolute fold change ≥2. Shared protein or gene intersections across groups were visualized using UpSet plots [85, 86]. Transcriptional Component Analysis (TCA) was applied to decompose gene expression variability into independent biological components across conditions. Gene Set Enrichment Analysis (GSEA) was performed to identify enriched pathways or biological processes using curated gene sets from MSigDB. All raw and processed data are available through repositories listed in the manuscript.

### Functional enrichment analysis

To identify biological processes overrepresented in the detected protein sets, Gene Ontology (GO) enrichment analysis was conducted using the clusterProfiler package [87, 88] for the “*Biological Process*”, “*Cellular Components*” and “*Molecular function*” ontologies. Results were corrected for multiple testing using the Benjamini–Hochberg method, and terms with adjusted *p-values* ≤ 0.05 were considered statistically significant.

### Repositories

RNA and protein data are stored in the following repositories:

- Caspase-3 Control of RNA Splicing and Mitochondrial Dynamics in Microglia during Parkinson’s Disease [RNA-Seq]. GEOGSE306116.
- Caspase-3 Control of RNA Splicing and Mitochondrial Dynamics in Microglia during Parkinson’s Disease [Array], GEOGSE304289
- Whole cell proteomics of microglia isolated from sustantia nigra upon 96 h treatment with saline or MPTP.PRIDEPXD066994.
- Analysis of proteins interacting with cleaved caspase-3 under basal conditions in microglia cell line BV2.PRIDEPXD066997.

### Statistical analysis

GraphPad Prism 9.0.0 was used for all statistical analyses. Normality of residuals was assessed using the Shapiro–Wilk test. Homogeneity of variances was evaluated using Brown–Forsythe and Bartlett’s tests. When comparing more than two variables, one-way, two-way or three-way ANOVA was used, with Welch’s and/or Brown–Forsythe corrections applied in cases of heteroscedasticity.

For pairwise comparisons, parametric paired or ratio paired *t*-tests were used, and Wilcoxon matched-pairs signed-rank tests were applied when normality was not met. Spearman’s test was used to assess heteroscedasticity in certain models. A significance threshold of *p* < 0.05 was used throughout.

Sample sizes were determined based on a priori power calculations assuming an α of 0.05 and a power of 80% to detect a biologically relevant effect size estimated from pilot experiments and published literature. Variance estimates were derived from preliminary data. The final sample size was sufficient to detect the specified effect using the statistical tests described.

For animal studies, sample sizes were chosen based on prior experience with these experimental models and on published studies using comparable approaches, which have demonstrated sufficient power to detect biologically meaningful differences. All experiments were independently replicated to ensure robustness of the findings.

## Supplementary information

Supplementary Figures 1–6 and list of reagents and sequences

Immunoblots

## Data Availability

This study did not generate new unique reagents. Further information and requests for resources and reagents should be directed to and will be fulfilled by the Lead Contact, Miguel Ángel Burguillos (miguel.burguillos@csic.es).
