# Supplementary material for: Caspase-3 regulates RNA splicing and mitochondrial dynamics in microglia during Parkinson’s disease
Source: Cell Death Dis. 2026 Aug 4;17(1):834. doi: 10.1038/s41419-026-09141-x (PMC13614962; doi:10.1038/s41419-026-09141-x)
Supplement: Supplementary file 1 [file 41419_2026_9141_MOESM1_ESM.pdf]

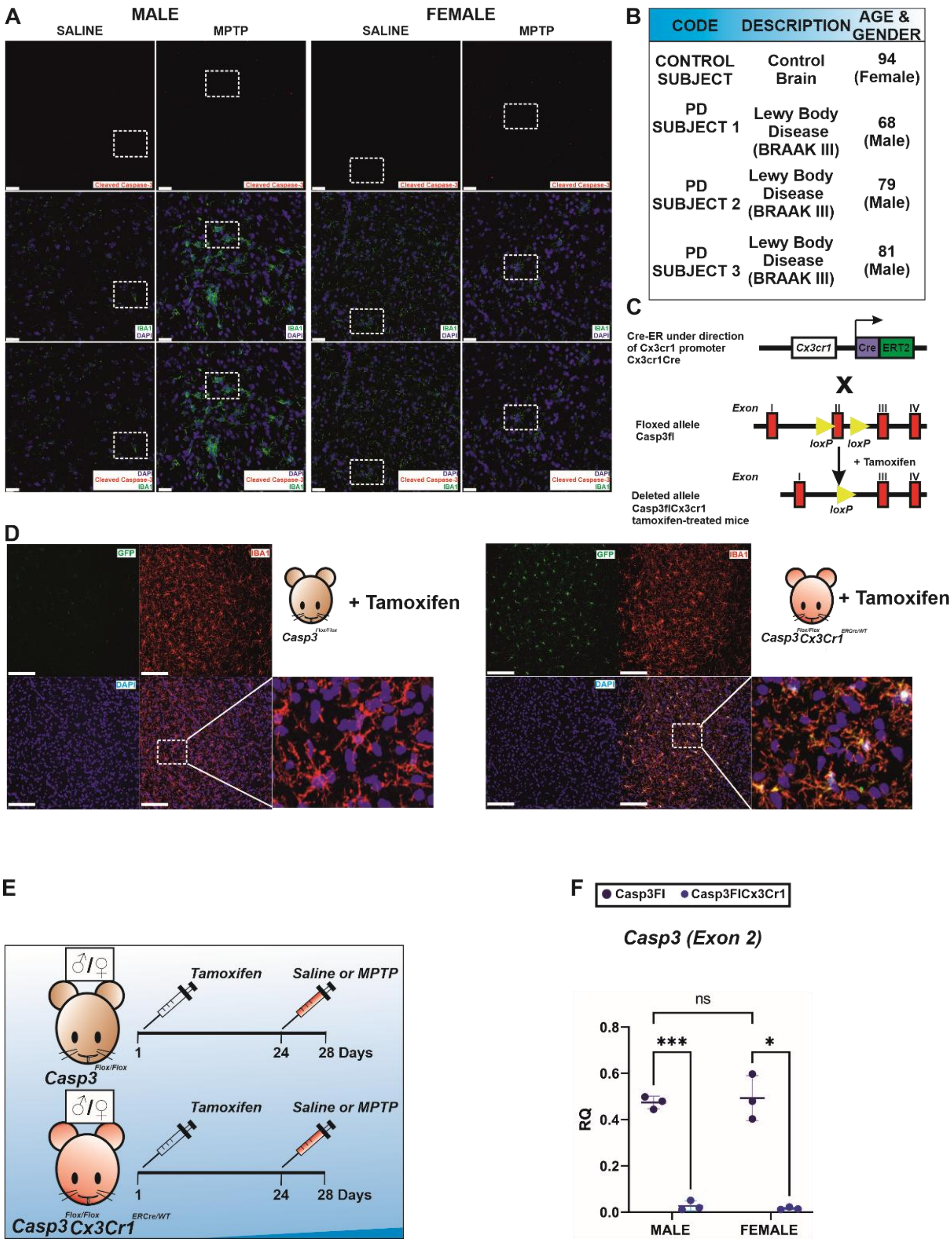

Supplementary Figure S1. Validation of Caspase-3 Deletion Efficiency model, related to Figure 1

(A) Full confocal images corresponding to insets shown in Figure 1A; dashed rectangles indicate regions magnified. Scale bar, 25 µm. (B) Table including the age and gender of the control and severity of PD subjects in Figure 1B. (C) Schematic of the tamoxifen-inducible Cre-loxP system targeting exon 2 of Casp3. (D) Representative confocal images showing GFP (green), IBA1 (red), and DAPI (blue) in *Casp3*$^{flox/flox}$ and *Casp3*$^{flox/flox}$*Cx3cr1*$^{CreERT2/WT}$ mice after tamoxifen induction. Scale bar, 100 µm. (E) Schematic of tamoxifen induction and MPTP/saline treatment in *Casp3*$^{flox/flox}$ and *Casp3*$^{flox/flox}$*Cx3cr1*$^{CreERT2/WT}$ male/female mice. (F) RT-qPCR analysis of Casp3 mRNA levels using exon 2-specific primers in microglia isolated from the SN of tamoxifen-treated *Casp3*$^{flox/flox}$ and *Casp3*$^{flox/flox}$*Cx3cr1*$^{CreERT2/WT}$ mice. Each dot represents one biological replicate (n = 3 mice per group).

Statistics: one-way ANOVA with Dunnett's T3 *post hoc* test (F). *$^{*}p < 0.05$, $^{***}p < 0.001$, ns=* statistically non-significant.

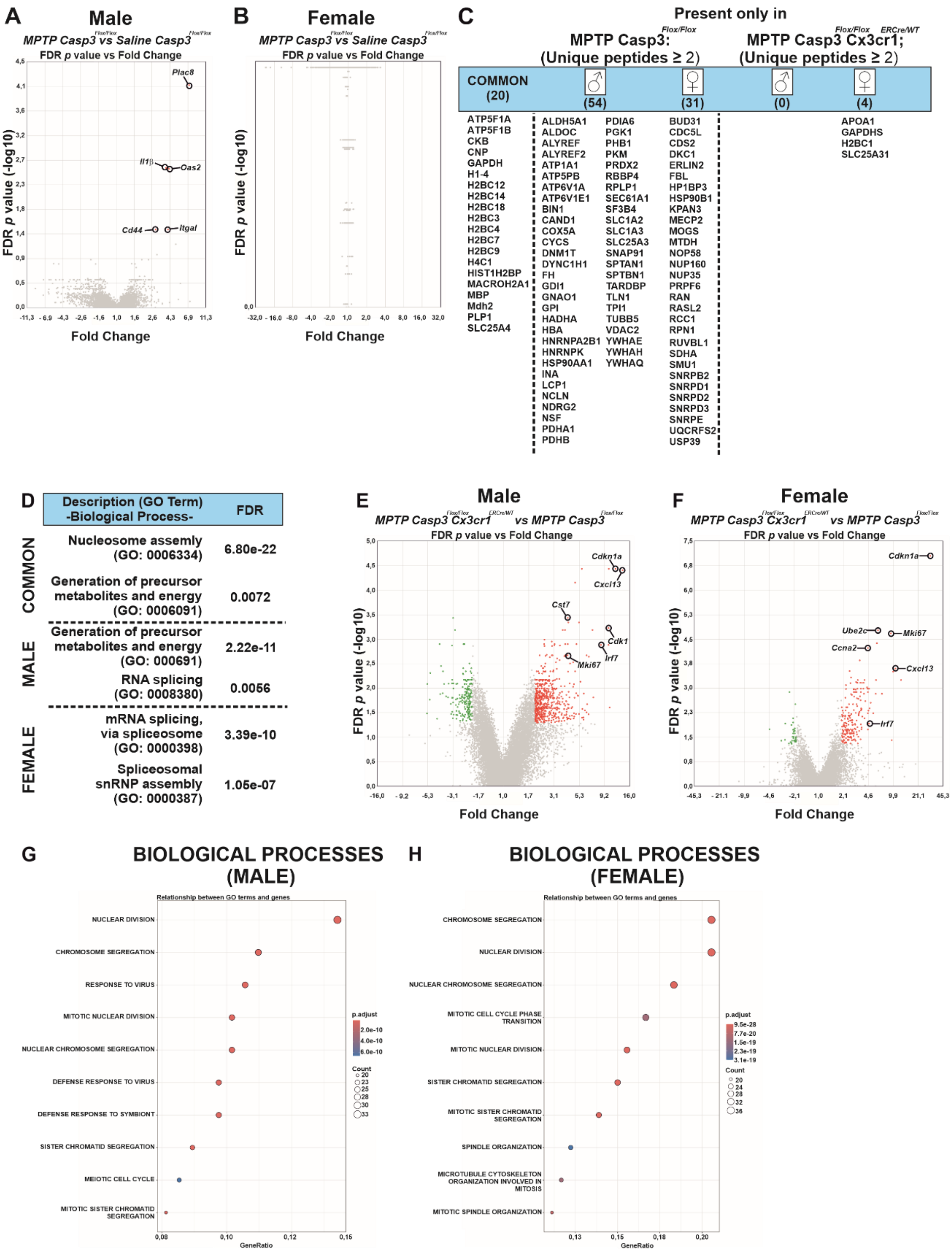

Supplementary Figure S2. Differential Gene Expression and Protein Profiling in Microglia Following MPTP Treatment and in the absence of caspase-3, related to Figure 2

(A, B) Volcano plots showing differentially expressed genes (FDR < 0.05) in microglia isolated from the substantia nigra (SN) of male (A) and female (B) *Casp3$^{flox/flox}$* mice treated with MPTP versus saline (n = 3 mice per group). (C) Protein presence/absence table showing unique and

shared proteins identified by proteomic analysis in microglia from male and female MPTP-treated *Casp3*$^{flox/flox}$ and *Casp3*$^{flox/flox}$*Cx3cr1*$^{CreERT2/WT}$ mice (related to Figure 2E). Only proteins detected in both replicates and annotated with ≥2 unique peptides are shown (n = 2 mice per group). (D) GO terms enriched among proteins listed in (C). (E, F) Volcano plot for differentially expressed genes (FDR < 0.05) in male (E) and female (F) *Casp3*$^{flox/flox}$*Cx3cr1*$^{CreERT2/WT}$ MPTP microglia versus *Casp3*$^{flox/flox}$ microglia MPTP (n = 3 mice per group). (G, H) Gene Ontology (GO) enrichment dot plot for differentially expressed genes (FDR < 0.05) in male (G) and female (H) *Casp3*$^{flox/flox}$*Cx3cr1*$^{CreERT2/WT}$ MPTP microglia versus *Casp3*$^{flox/flox}$ microglia MPTP (n = 3 mice per group)

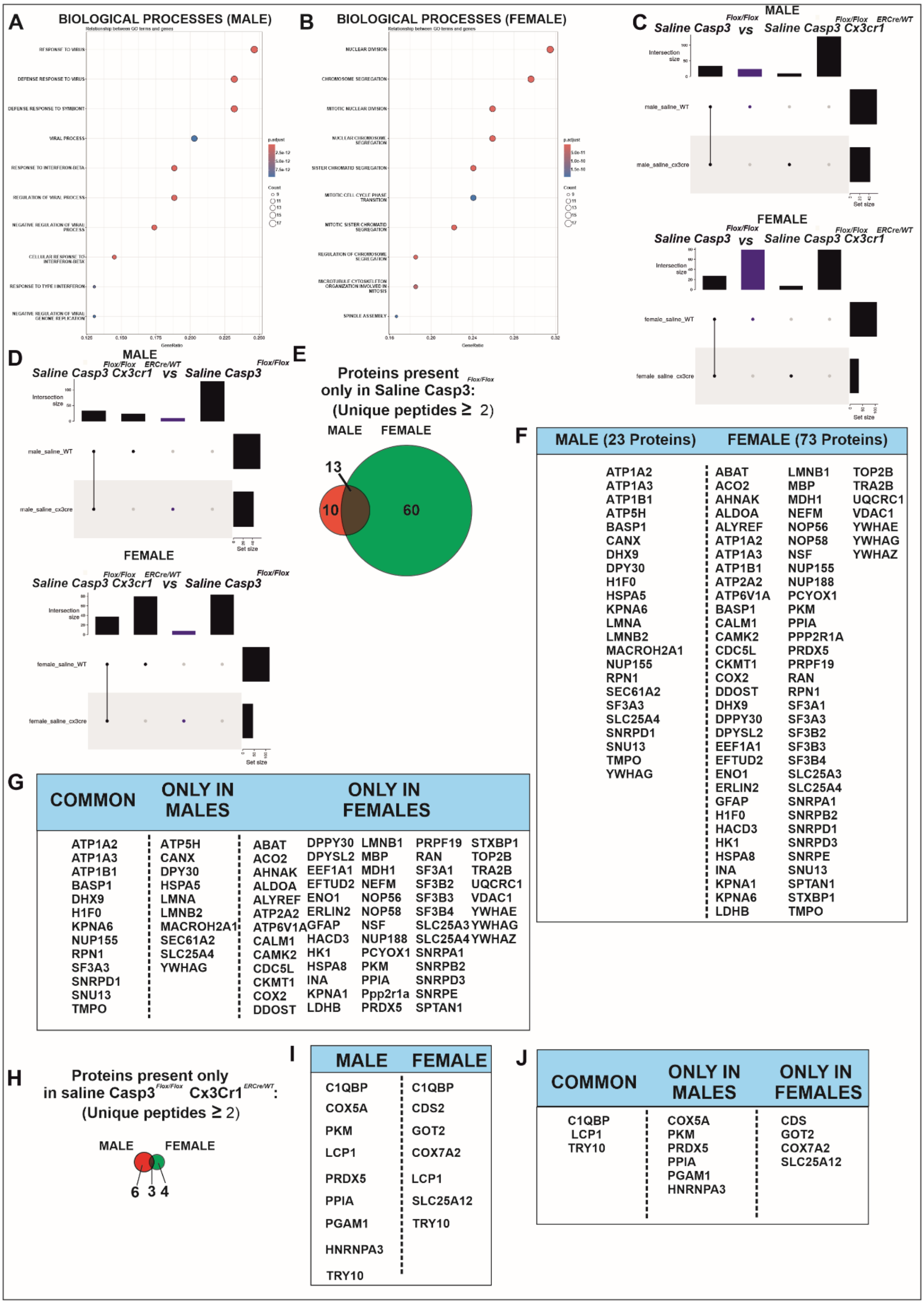

Supplementary Figure S3. Differential Gene Expression and Protein Profiling in Microglia Following Caspase-3 Deletion under Saline Conditions, related to Figure 3

(A, B) Gene Ontology (GO) enrichment dot plots for differentially expressed genes (FDR < *0.05*) in microglia isolated from the substantia nigra (SN) of male (A) and female (B) *Casp3$^{flox/flox}$Cx3cr1$^{CreERT2/WT}$ versus Casp3$^{flox/flox}$* mice under saline condition (n = 3 mice per group). (C, D) Upset plots showing the number of proteins identified via mass spectrometry in

male and female microglia from *Casp3*$^{flox/flox}$ (C) and *Casp3*$^{flox/flox}$*Cx3cr1*$^{CreERT2/WT}$ (D) and under saline treatment. Only proteins detected in both replicates and annotated with ≥2 unique peptides are included (n = 2 mice per group). (E) Venn diagram showing shared and unique proteins between male and female microglia in *Casp3*$^{flox/flox}$ mice under saline condition. (F) Table listing proteins enriched in male and female *Casp3*$^{flox/flox}$ microglia under saline condition. (G) Same protein list as in (F), categorized by shared, male-specific, and female-specific proteins. (H) Venn diagram showing shared and unique proteins between male and female microglia in *Casp3*$^{flox/flox}$*Cx3cr1*$^{CreERT2/WT}$ mice under saline condition. (I) Table listing proteins enriched in male and female *Casp3*$^{flox/flox}$*Cx3cr1*$^{CreERT2/WT}$ microglia under saline condition. (J) Same protein list as in (I), categorized by shared, male-specific, and female-specific protein.

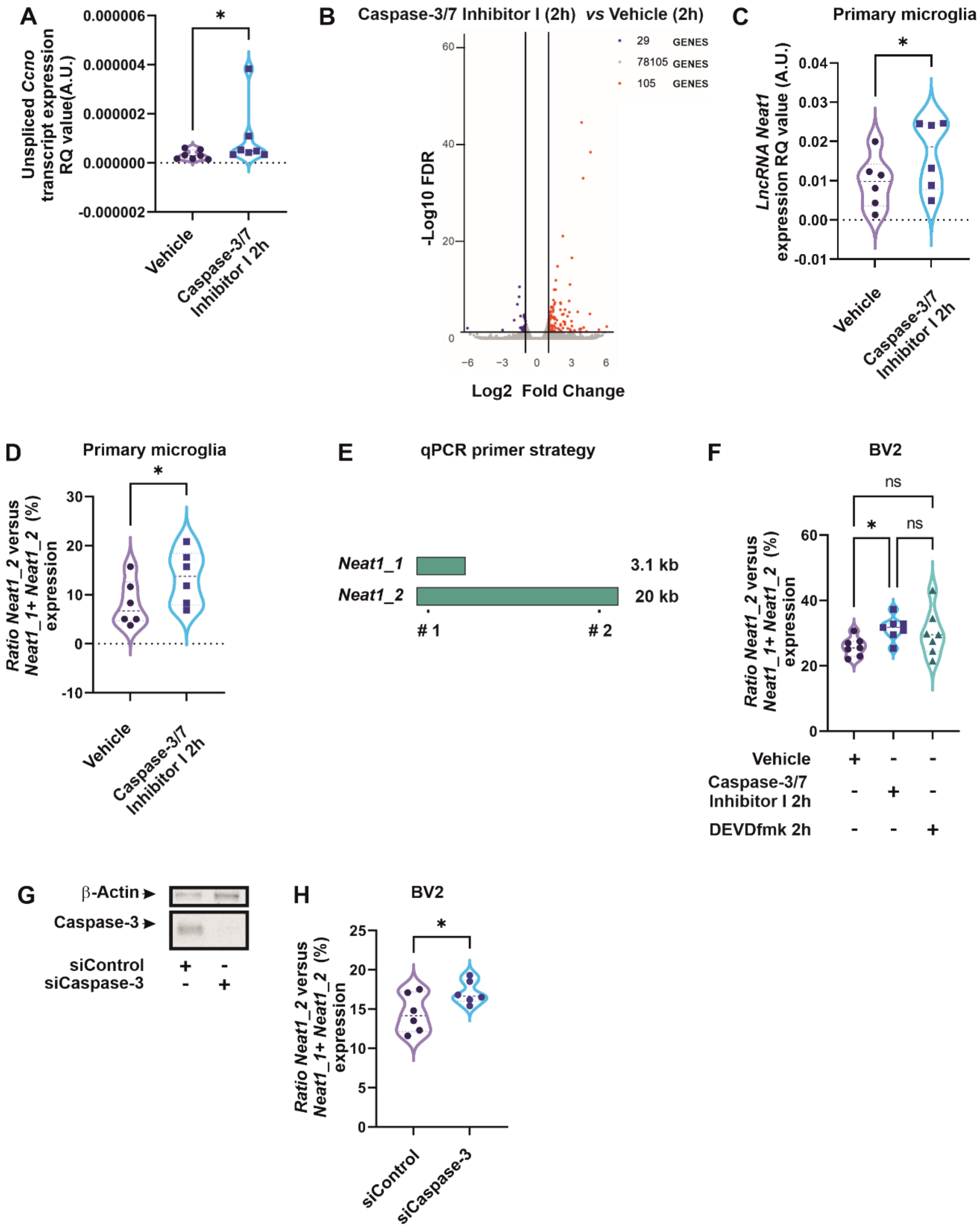

Supplementary Figure S4. Caspase-3 inhibition induces Ccno intron retention and modulates NEAT1 expression and paraspeckle-associated features in primary microglia, related to Figure 4.

(A) Validation of Ccno intron retention following 2 h allosteric caspase-3/7 inhibitor I, assessed by intron–exon junction qPCR and expressed relative to vehicle-treated controls in primary microglia cells. (B) Volcano plot showing differential gene expression after 2 h of allosteric caspase-3/7 inhibitor I (FDR<0.05). (C) RT-qPCR analysis of mRNA expression levels of LncRNA *Neat 1* (n=7 independent experiments). (D) RT-qPCR analysis of the % of the ratio Neat1_2 after 2 h of allosteric caspase-3/7 inhibitor I in primary microglia cells (n=6 independent experiments). (E) Schematic representation of Neat1 isoforms, indicating the short (Neat1_1) and long (Neat1_2) transcripts, including the strategy for primer design to differentiate between both

isoforms. (F) RT-qPCR analysis of the % of the ratio Neat1_2 after 2 h of allosteric caspase-3/7 inhibitor I and DEVDfmk in BV2 cells (n=7 independent experiments). (G) Representative immunoblot showing successful silencing of caspase-3 in BV2 cells. (H) RT-qPCR analysis of the % of the ratio Neat1_2 upon caspase-3 silencing in BV2 cells by qPCR (n=6 independent experiments).

Statistics: Paired Wilcoxon signed-rank test. (A, C); Paired t-Test (D, H); one-way repeated-measures ANOVA followed by Tukey's multiple comparisons test (F); paired-t test (H) *$p < 0.05$.

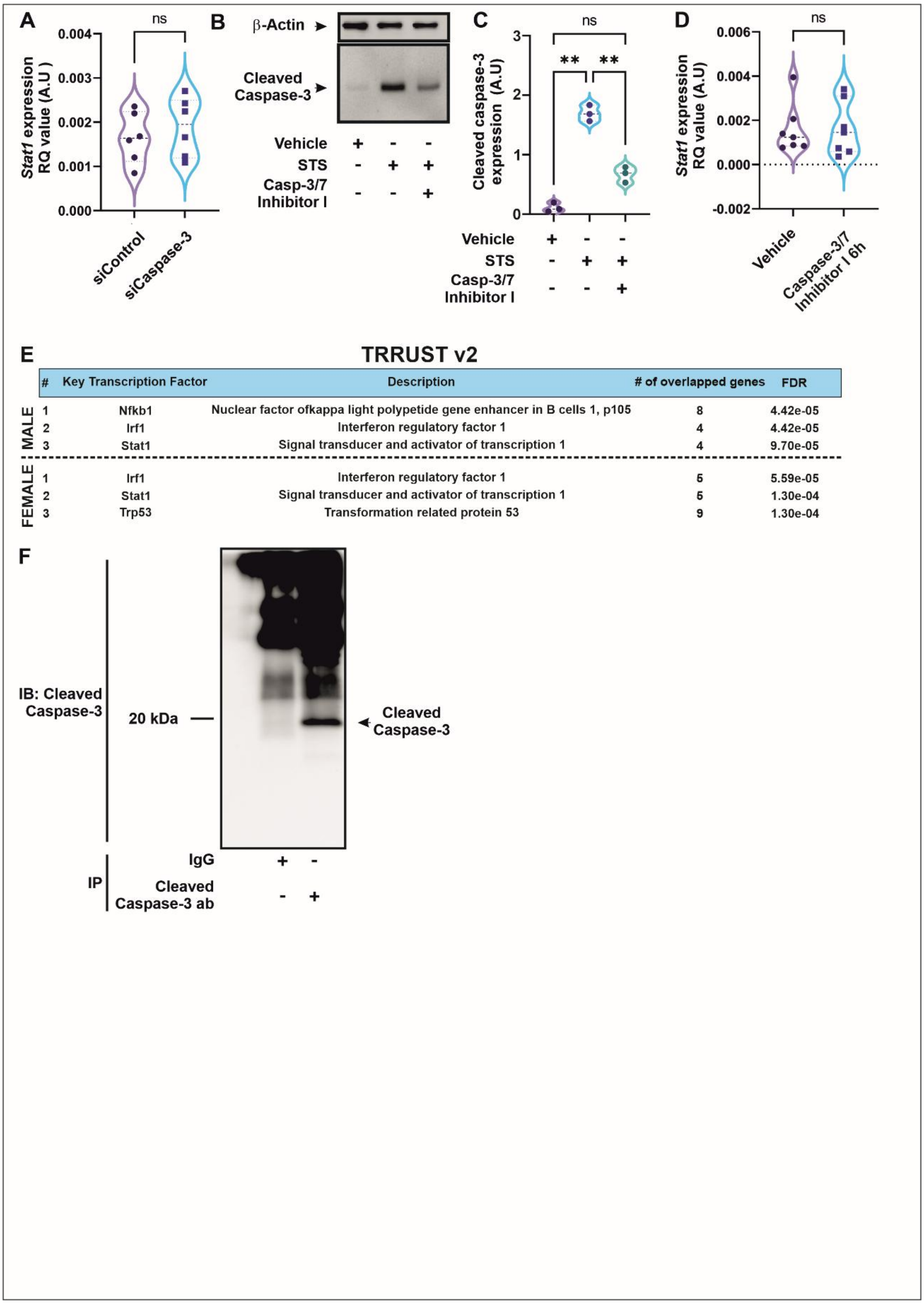

Supplementary Figure S5. Caspase-3 Deficiency Does Not Alter Stat1 mRNA Expression and Validation of Caspase-3/7 Inhibition in BV2 Cells, related to Figure 5

(A) RT-qPCR analysis of *Stat1* mRNA expression following siRNA-mediated knockdown of caspase-3 (n = 6 independent experiments). (B) Representative Immunoblot and analysis of the

effect of 4h cotreatment of caspase-3 /7 inhibitor I with staurosporine (STS) over caspase-3 activation (n = 3 independent experiments) (C). (D) RT-qPCR analysis of *Stat1* mRNA levels following a 6-hour treatment with caspase-3/7 inhibitor I (n = 7 independent experiments). (E) Predicted transcription factors (TFs) with associated FDR values responsible for differentially expressed genes in male and female caspase-3-deficient microglia under saline condition, identified using the TRRUST v2 tool. (F) Immunoprecipitation using the cleaved caspase-3 antibody employed in mass spectrometry analysis of BV2 cells under basal conditions.

Statistics: one-way ANOVA with Tukey's *post hoc* test (C); Paired T-test (A, D) **$p < 0.01$, *ns*= statistically non-significant.

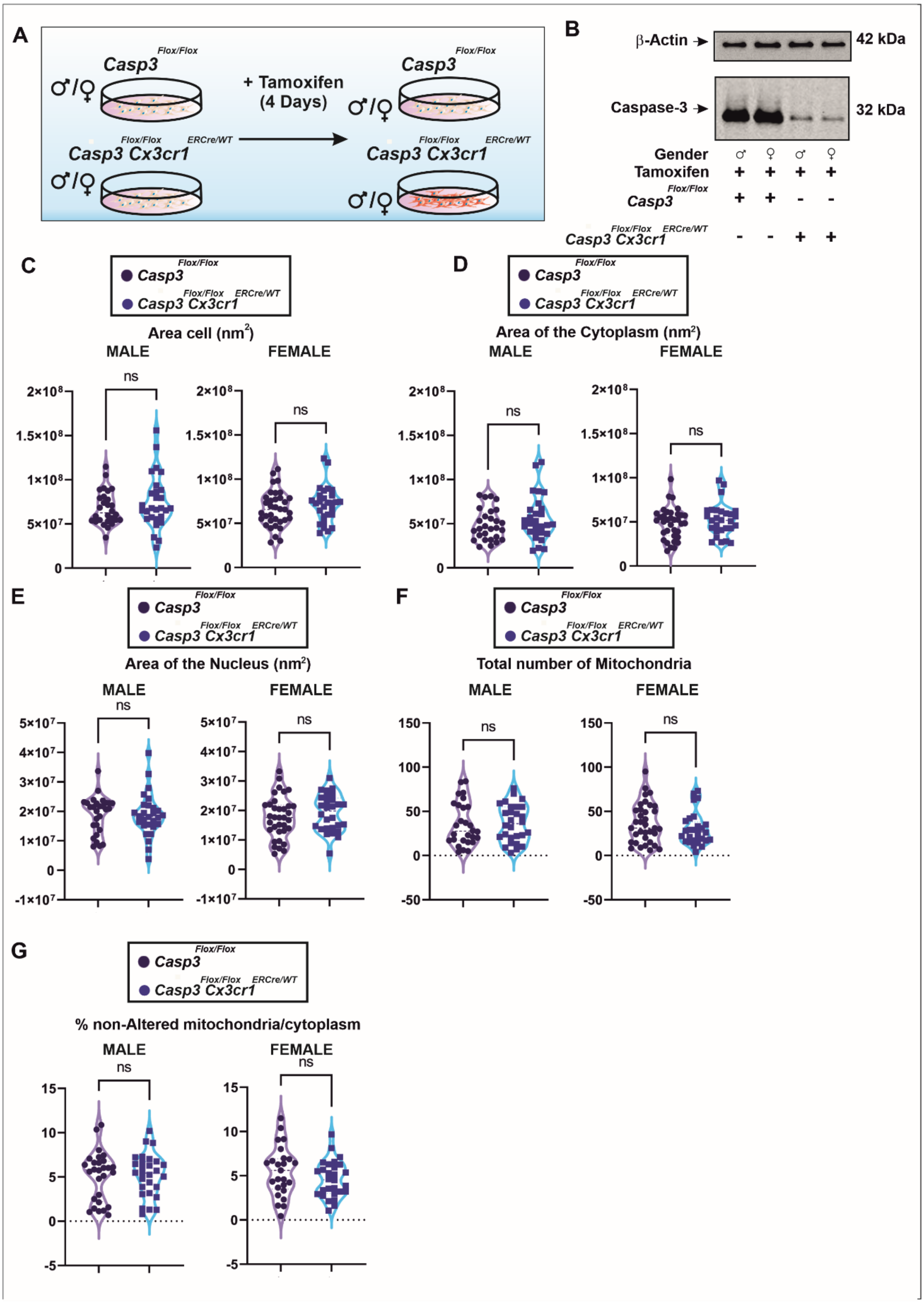

Supplementary Figure S6. Caspase-3 Deficiency Does Not Alter Mitochondrial Size or Number in Gender-Segregated Primary Microglia, related to Figure 7

(A) Schematic representation of the tamoxifen treatment timeline in gender-segregated primary microglia. (B) Representative immunoblot showing efficient deletion of caspase-3 only in *Casp3*$^{flox/flox}$*Cx3cr1*$^{CreERT2/WT}$ primary microglia following tamoxifen treatment. (C–E) Quantification of cellular compartments in male and female cultures, including total cell area (C),

cytoplasmic area (D), and nuclear area (E). (F) Quantification of total number of mitochondria per cell in male and female microglia cultures. (G) Quantification of cytoplasmic area occupied by non-altered mitochondria in male and female cultures. Data in (C–G) represent mean ± SD from three independent experiments, with 10 cells analysed per treatment group per experiment.

Statistics: Unpaired T-test (C -FEMALE-, E -MALE and FEMALE-, G -FEMALE-); Mann–Whitney U test (C -MALE-, D -MALE and FEMALE-, F – MALE and FEMALE-, G -MALE-). *ns*= statistically non-significant

Full list of reagents, antibodies and cell lines, animal strains and bioinformatic program used (including identifiers):

REAGENT or RESOURCE	SOURCE	IDENTIFIER
Antibodies		
β-Actin Antibody	Sigma-Aldrich	Cat#A1978; RRID:AB_476692
Caspase-3 Antibody	Cell Signaling Technology	Cat# 9662; RRID:AB_331439
Cleaved Caspase-3 Antibody	Cell Signaling Technology	Cat# 9661; RRID:AB_2341188
Cleaved Caspase-3 Antibody (BSA and Azide free)	Cell Signaling Technology	Cat# 94530SF; RRID:AB_3076239
Donkey anti-Goat IgG (H+L) Cross-Adsorbed Secondary Antibody, Alexa Fluor™ 546	Thermo Fisher Scientific	Cat# A-11056; RRID:AB_2534103
Donkey anti-Rabbit IgG (H+L) Highly Cross-Adsorbed Secondary Antibody, Alexa Fluor™ 488	Thermo Fisher Scientific	Cat# A-21206; RRID:AB_2535792
Donkey anti-Goat IgG (H+L) Highly Cross-Adsorbed Secondary Antibody, Alexa Fluor™ Plus 647	Thermo Fisher Scientific	Cat# A32849; RRID:AB_2762840
Goat Anti-Rabbit IgG Antibody (H+L), Peroxidase	Vector Laboratories	Cat# PI-1000-1; RRID:AB_2916034
dsRNA Antibody, clone rJ2 Antibody	Millipore	Cat# MABE1134; RRID:AB_2819101
GFP Antibody	Novus	Cat# NB100-1770; RRID:AB_10128178
Horse Anti-Rabbit IgG (H+L) Biotinylated	Vector Laboratories	Cat# BA-1100; RRID:AB_2336201
Horse Anti-Mouse IgG Antibody (H+L), Peroxidase	Vector Laboratories	Cat# PI-2000-1; RRID:AB_2336177
Iba-1 Antibody	Abcam	Cat# ab289874; RRID:AB_2942069
Iba-1 Antibody	FUJIFILM Wako Pure Chemical Corporation	Cat# 019-19741; RRID:AB_839504
Rabbit IgG Isotype Control Antibody	Thermo Fisher Scientific	Cat# 02-6102; RRID:AB_2532938
STAT1 Antibody	Cell Signaling Technology	Cat# 14994: RRID:AB_2737027
Tyrosine Hydroxylase Antibody (TH) Antibody	Sigma-Aldrich	Cat# T8700; RRID:AB_1080430)
Bacterial and virus strains		

Biological samples		
Human *substantia nigra* belonging to PD patients and Control	University Hospital Clinic (Barcelona)/IDIBAPS	Ethical committee number 0025-N-21
Chemicals, peptides, and recombinant proteins		
Glial-Mag	OZ Biosciences	Cat# KGL0250
Donkey serum	Sigma Aldrich	Cat# 9663
Horse serum	Sigma Aldrich	Cat# H1270
Target Retrieval Solution, Citrate pH 6 (x 10)	Dako	Cat# S 2369
ProLong Diamond Antifade Mountant	Thermo Fisher Scientific	Cat# P36965
TRI Reagent®	Sigma Aldrich	Cat# T9424
Caspase-3/7 Inhibitor I (allosteric)	Cayman Chemical	Cat# 14464
Caspase-3/7 Inhibitor (DEVDfmk; irreversible)	MedChemExpress	Cat# HY-12466
Autofluorescence Eliminator Reagent	Millipore	Cat# 2160
DPX mounting medium	Sigma Aldrich	Cat# 06522
RevertAid First Strand cDNA Synthesis Kit	ThermoFisher Scientific	Cat# K1622
SensiFAST™ SYBR® No-ROX Kit	Meridian	Cat# BIO-98005
Taqman Universal PCR Master Mix	ThermoFisher Scientific	Cat# 4304437
4,6-Diamidino-2-phenylindole dihydrochloride (DAPI)	Sigma-Aldrich	Cat# D9542
DAB Substrate kit	VectorLabs	Cat#SK4100
VECTASTAIN® Elite® ABC-HRP Kit, Peroxidase (Standard)	VectorLabs	Cat# PK-6100
Seahorse XF Calibrant Solution	Agilent	Cat# 100840-000
Seahorse XF DMEM Medium, pH 7.4	Agilent	Cat# 103575-100
Seahorse XF 100mM Pyruvate Solution	Agilent	Cat# 103578-100
Seahorse XF 1M Glucose Solution	Agilent	Cat# 103577-100
L-Glutamine 200mM Solution	Sigma Aldrich	Cat# 59202C
Oligomycin	Agilent	Included in kit Cat# 103591-100
Rotenone + Antimycin A (Rot/AA)	Agilent	Included in kit Cat# 103591-100
XFe96/XF Pro Cell Culture Microplate	Agilent	Cat# 103794-100
Pierce™ ECL Western blotting Substrate	ThermoFisher Scientific	Cat# 32106
Pierce™ BCA Protein Assay Kit	ThermoFisher Scientific	Cat# 23225
Critical commercial assays		
AllPrep DNA/RNA Mini Kit (50)	Qiagen	Cat# 80204
CD11b MicroBeads, human and mouse	Miltenyi Biotec	Cat#130-097-142
GeneChip™ 3' IVT Pico Kit	Thermo Fisher Scientific	Cat#902789
Clariom™ S Assay, mouse	Thermo Fisher Scientific	Cat# 902930
Protease Inhibitor Cocktail	Sigma Aldrich	Cat# P8340
Phosphatase Inhibitor Cocktail 2	Sigma Aldrich	Cat# P5726
Phosphatase Inhibitor Cocktail 3	Sigma Aldrich	Cat# P0044

Uranyl Acetate, Reagent, A.C.S.	Electron Microscopy Sciences	Cat# 2240
Sodium cacodylate trihydrate ≥99	Start Bioscience	Cat# 12300
Crystalline Osmium Tetroxide 99.95%	Start Bioscience	Cat# 19100
Spurr's Low Viscosity Embedding Media Kit	Electron Microscopy Sciences	Cat# 14300
Gilder Standard Hexagonal Mesh Grids	Electron Microscopy Sciences	Cat# G200H-CU
Glutaraldehyde 25% ME	Aname	Cat# 16220
Formaldehyde, solution	Scharlab	Cat# FO0013005P
Deposited data		
Caspase-3 Control of RNA Splicing and Mitochondrial Dynamics in Microglia during Parkinson's Disease [RNA-Seq]	GEO	GSE306116
Caspase-3 Control of RNA Splicing and Mitochondrial Dynamics in Microglia during Parkinson's Disease [Array]	GEO	GSE304289
Whole cell proteomics microglia isolated from sustantia nigra upon 96h treatment with saline or MPTP	PRIDE	PXD066994
Analysis of proteins interacting with cleaved caspase-3 under basal conditions in microglia cell line BV2	PRIDE	PXD066997
Experimental models: Cell lines		
Mouse: BV2. Passage ≤ 25 (Female). Checked for mycoplasma contamination but not authenticated recently	Bertrand Joseph′s lab (Shen et al., 2016)	RRID: CVCL_0182
Experimental models: Organisms/strains		
Mouse: B6N.129S1-Casp3tm1Flv/J (Male and Female)	Jackson Laboratories	Cat#:006233; RRID: IMSR_JAX:006233
Mouse: CX3CR1CreERT2/WT: B6.129P2(Cg) Cx3cr1tm2.1(cre/ERT)Litt/WganJ (Male and Female)	Jackson Laboratories	Cat#:021160; RRID: IMSR_JAX:021160
Mouse: BALB/c (Male and Female)	Centro de Experimentación Animal Óscar Pintado (CITIUS)	RRID: MGI:2161072
Oligonucleotides		
Taqman® Gene Expression Array Ifnb1	ThermoFisher Scientific	Cat# Mm00439552_s1
Taqman® Gene Expression Array PGK1	ThermoFisher Scientific	Cat# Mm00435617_m1
siGENOME Non-Targeting siRNA Pool #1	Horizon Discovery	Cat# D-001206-13-05
ON-TARGETplus SMARTpool siRNA Caspase-3 Pool of 4 siRNAs	Horizon Discovery	Cat# L-043042-00-0005
Software and algorithms		
Fiji ImageJ	http://fiji.sc/	RRID:SCR_002285
GraphPad Prism 9	https://www.graphpad.com/scientific-software/prism/	RRID:SCR_002798

Leica Application Suite X	https://www.leica-microsystems.com/products/microscope-software/details/product/leica-las-x-ls/	RRID:SCR_013673
Transcriptome Analysis Console	https://www.thermofisher.com/es/es/home/life-science/microarray-analysis/microarray-analysis-instruments-software-services/microarray-analysis-software/affymetrix-transcriptome-analysis-console-software.html	RRID:SCR_016519
Gene Set Enrichment Analysis (GSEA)	http://www.broadinstitute.org/gsea/	RRID:SCR_003199
Transcriptional Regulatory Relationships Unrevealed by Sentence based Text mining database (TRUSTT v2)	https://www.grnpedia.org/trrust/	RRID:SCR_022554
newCAST stereology software	https://visiopharm.com/visiopharm-digital-image-analysis-software-features/stereology/	RRID:SCR_014570
bcl2fastq	https://support.illumina.com/sequencing/sequencing_software/bcl2fastq-conversion-software.html	RRID:SCR_015058
HISAT2	http://ccb.jhu.edu/software/hisat2/index.shtml	RRID:SCR_015530
Deeptools	https://deeptools.readthedocs.io/en/develop/	RRID:SCR_016366
edgeR	http://bioconductor.org/packages/edgeR/	RRID:SCR_012802
DEXSeq	http://bioconductor.org/packages/release/bioc/html/DEXSeq.html	RRID:SCR_012823
R Project for Statistical Computing	http://www.r-project.org/	RRID:SCR_001905
clusterProfiler	http://bioconductor.org/packages/release/bioc/html/clusterProfiler.html	RRID:SCR_016884
org.Mm.eg.db	https://bioconductor.org/packages/org.Mm.eg.db/	RRID:SCR_023488

ggplot2	https://cran.r-project.org/web/packages/ggplot2/index.html	RRID:SCR_014601
ComplexHeatmap	https://bioconductor.org/packages/release/bioc/html/ComplexHeatmap.html	RRID:SCR_017270
Entrez Gene	http://www.ncbi.nlm.nih.gov/gene	RRID:SCR_002473

Primer list (including sequence)

Primer name and purpose		
Primers for Actb (mouse) for RT-qPCR	Forward primer (5´→3´): CCACACCCGCCACCAGTTCG	Reverse primer (5´→3´): CCCATTCCCACCATCACACC
Primers for Casp3 -exon2- (mouse) for RT-qPCR	Forward primer (5´→3´): GAGCTTGGAACGGTACGCTAA	Reverse primer (5´→3´): GAGTCCACTGACTTGCTCCC
Primers for Irf7 (mouse) for RT-qPCR	Forward primer (5´→3´): CCCATCTTCGACTTCAGCAC	Reverse primer (5´→3´): TGCCCAAAACCCAGGTAGAT
Primers for Stat1 (mouse) for RT-qPCR	Forward primer (5´→3´): TTTGACAGTATGATGAGCAC	Reverse primer (5´→3´): AGCAAATGTGATGCTCTTTC
Primers for LncRNA Neat 1 (#1) (mouse) for RT-qPCR	Forward primer (5´→3´): TTGGGACAGTGGACGTGTGG	Reverse primer (5´→3´): TCAAGTGCCAGCAGACAGCA
Primers for LncRNA Neat 1 (#2) (mouse) for RT-qPCR	Forward primer (5´→3´): GCTCTGGGACCTTCGTGACTCT	Reverse primer (5´→3´): CTGCCTTGGCTTGGAAATGTAA
Primers for Ccno spanning exon 1–intron 1 junction (mouse) for RT-qPCR	Forward primer (5´→3´): CACAAGTGAGGCGCTGGCAAGA	Reverse primer (5´→3´): TGGAGAGAGCGAGCTAGCCTGG
Primers for Casp3 floxed allele (A) for Genotyping	(5´→3´):GAGCCTTCATAGGGGTGCAA	
Primers for Casp3 floxed allele (B) for Genotyping	(5´→3´):GGGGAGCAGAGGGAATAAAG	
Primers for Casp3 floxed allele (C) for Genotyping	(5´→3´):CATAGAATCCCAAGCCAGGA	
Primers for Cx3cr1 Cre Common allele for Genotyping	(5´→3´):AAGACTCACGTGGACCTGCT	
Primers for Cx3cr1 Cre Wild Type allele for Genotyping	(5´→3´):AGGATGTTGACTTCCGAGTTG	
Primers for Cx3cr1 Cre Mutant allele for Genotyping	(5´→3´):CGGTTATTCAACTTGCACCA	

siRNA sequences from SMART POOL siRNAs (Company: Horizon Discoveries)

**siGENOME Non-Targeting siRNA pool #1**	**Cat# D-001206-13-05**
siRNA sequence 1	UAGCGACUAAACACAUCAA
siRNA sequence 2	UAAGGCUAUGAAGAGAUAC
siRNA sequence 3	AUGUAUUGGCCUGUAUUAG
siRNA sequence 4	AUGAACGUGAAUUGCUCAA
**ON-TARGETplus SMARTpool siRNA Caspase-3 Pool of 4 siRNAs**	**Cat# L-043042-00-0005**
siRNA sequence 1	GAAAUGGGCAUAUGCAUAA
siRNA sequence 2	CAACGGAAUUCGAGUCCUU
siRNA sequence 3	GGAUAGUGUUUCUAAGGAA
siRNA sequence 4	CGCACAAGCUAGAAUUUAU
